# The Genomic Legacy of the Transatlantic Slave Trade in the Yungas Valley of Bolivia

**DOI:** 10.1371/journal.pone.0134129

**Published:** 2015-08-11

**Authors:** Tanja Heinz, Jorge Mario Cárdenas, Vanesa Álvarez-Iglesias, Jacobo Pardo-Seco, Alberto Gómez-Carballa, Carla Santos, Patricia Taboada-Echalar, Federico Martinón-Torres, Antonio Salas

**Affiliations:** 1 Unidade de Xenética, Instituto de Ciencias Forenses and Departamento de Anatomía Patolóxica e Ciencias Forenses, Facultade de Medicina, Universidade de Santiago de Compostela, Santiago de Compostela, Galicia, Spain; 2 Grupo de Investigación en Genética, Vacunas, Infecciones y Pediatría (GENVIP), Hospital Clínico Universitario and Universidade de Santiago de Compostela (USC), Santiago de Compostela, Galicia, Spain; 3 Translational Pediatrics and Infectious Diseases, Department of pediatrics, Department of Pediatrics, Hospital Clínico Universitario de Santiago, Santiago de Compostela, Galicia, Spain; University of Florence, ITALY

## Abstract

During the period of the Transatlantic Slave Trade (TAST) some enslaved Africans were forced to move to Upper Peru (nowadays Bolivia). At first they were sent to Potosí, but later to the tropical Yungas valley where the Spanish colonizers established a so-called “hacienda system” that was based on slave labor, including African-descendants. Due to their isolation, very little attention has been paid so far to ‘Afro-Bolivian’ communities either within the research field of TAST or in genetic population studies. In this study, a total of 105 individuals from the Yungas were sequenced for their mitochondrial DNA (mtDNA) control region, and mitogenomes were obtained for a selected subset of these samples. We also genotyped 46 Ancestry Informative Markers (AIM) in order to investigate continental ancestry at the autosomal level. In addition, Y-chromosome STR and SNP data for a subset of the same individuals was also available from the literature. The data indicate that the partitioning of mtDNA ancestry in the Yungas differs significantly from that in the rest of the country: 81% Native American, 18% African, and 1% European. Interestingly, the great majority of ‘Afro-descendant’ mtDNA haplotypes in the Yungas (84%) concentrates in the locality of Tocaña. This high proportion of African ancestry in the Tocaña is also manifested in the Y-chromosome (44%) and in the autosomes (56%). In sharp contrast with previous studies on the TAST, the ancestry of about 1/3 of the ‘Afro-Bolivian’ mtDNA haplotypes can be traced back to East and South East Africa, which may be at least partially explained by the Arab slave trade connected to the TAST.

## Introduction

The Transatlantic Slave Trade (TAST) resulted in about 10.7 million slaves arriving in the New World in the period 1501–1866 [1]. During the peak of the TAST, in the second half of the 18^th^ century, on average of 60,000 enslaved Africans, reached the ports of America each year [1]. According to the TAST database (http://www.slavevoyages.org/), out of the total number of enslaved Africans who disembarked in the Americas between 1514 and 1866 (8,185,024), most were transported to the Caribbean (51.9%) and Brazil (38.9%), followed by mainland North America (3.8%), and the Spanish American mainland (3.3%). Historical documents indicate that most enslaved Africans who arrived to the Spanish American mainland originated from West-Central Africa and St. Helena (57.0%) or from Senegambia and the offshore Atlantic (19.4%) [1]; see S1 Text for further notes on the TAST.

Bolivia has a total population of about 10.2 million people (Instituto Nacional de Estadística de Bolivia—INE, http://www.ine.gob.bo/), dominated mainly by its indigenous population. ‘African descendant’ communities, however, can still be found, mainly in the provinces of Nor (North) and Sud (South) Yungas in the department of La Paz. The Bolivian Yungas is a tropical and subtropical moist forest eco-region with poor accessibility from the major cities. Here, ‘Afro-Bolivian’ communities remained mostly isolated after the agrarian reform in 1952. Today, there are several ‘Afro-Bolivian’ communities in the Nor Yungas (Tocaña, Mururata, Chijchipa, Dorado Chico, Coscoma, and Khala Kahla) and in the Sud Yungas (Chicaloma) [2]. Only in approximately the last twenty years ‘Afro-descendant’ people have migrated out from the Yungas. The category ‘Afro-Bolivians’ was only included in the national census for the first time in 2007 [3].

Not only in Bolivia itself [4], but also among international scholars of the TAST, ‘Afro-Bolivian’ people have either been neglected or not mentioned at all, possibly due to their small numbers when compared to other American countries and regions with a more evident African genetic heritage (e.g. Caribbean, Brazil, Colombia) [5–9].

Based on historical documentation, there are two main reasons why we would expect Senegambia to be one of the most important contributors to ‘Afro-Bolivians’. First, the TAST began in the Senegambia region, and, second, most of the slave deportations until the first decades of the 17^th^ century were mainly destined for Spanish colonies, especially Mexico, but also Lima (Peru) where slaves were further sold to Upper Peru (Bolivia), Chile or Quito [10]. However, Senegambia became gradually less important after the beginning of the 17^th^ century as other regions, especially West-Central Africa, became increasingly more important for European traders [1].

To the best of our knowledge, no detailed historical information is available that conclusively documents the African origins of modern-day ‘Afro-Bolivian’ communities. However, the earliest report of enslaved Africans in Potosí, Upper Peru, date to 1549 [11], indicating that slave displacement to Bolivia began early, and thus included the period when Senegambia was the major slave provider. Some sources indicate that Congo and Angola could also have contributed slaves to Bolivia [1,2]. A Congolese and Angolan origin can be supported by the presence in the Yungas of African surnames such as Angola or Maconde, which purportedly originated in Congo [12]. Furthermore, it was also documented in Charcas (Bolivia) that ethnic groups arriving in Upper Peru between 1650 and 1710, and which included principally females, could be traced back mainly to West and Central Africa, but also to Upper Guinea [13].

Studying genetics in the context of the TAST has confirmed some important facts about the trade and, therefore, underlined the reliability and convenience of genetics to investigate the past. Mitochondrial DNA (mtDNA) has been shown to be a suitable tool to trace back present-day ‘Afro-American’ haplotypes to broad geographic regions in Africa. In agreement with known historical phenomena, major mtDNA African contributions in present day American populations can be traced back to western and West-Central African regions [14–16]; whereas southeastern Africa contributed a minor proportion, especially to South America (e.g. see Chocó in Colombia, and the Garífunas in Honduras and Belize [8]). Similarly, Stefflova et al. [17] have shown that genetically distinct populations from Africa contributed unequally to the mtDNA gene pool of ‘African-descendant’ populations in North, Central and South America. For example, West / West-Central Africa contributed mostly to Caribbean populations and less, but still substantially, to populations in the United States and Brazil; whereas Southeastern Africa was observed to contribute principally to the Brazilian and Colombian gene pool [17]. However, tracing one’s own ancestors to a single ethnic group in Africa is problematic as many mtDNA haplogroups can be shared by various African ethnic groups [15,18,19].

Ancestry proportions measured by means of uniparental markers such as mtDNA, and also the Y chromosome, can further reflect gender-biased practices during the TAST. In other words, the New World was principally colonized by European males, which can be observed in an elevated European ancestry contribution on the Y chromosome, and a comparatively low European ancestry on the mtDNA. This has been observed in American countries, e.g. in the Brazilian population [9,20], in Bolivia [21], and in Argentina [22], but also in African colonies such as the Cape Verde islands, which were taken over by the Portuguese and converted to a sugar plantation [23].

In the most recent studies, the TAST has also been investigated using autosomal DNA markers. For example, Moreno-Estrada et al. [24] carried out a genome-wide study and found two major migration pulses from Africa into the Caribbean: one beginning around 1550, and the other one at the peak of the TAST at the end of the 18^th^ century. In agreement with historical records, these authors also showed by means of genomic segment length that, during the first migration pulse, the individuals mostly came from the Senegambia region, whereas during the second pulse most enslaved Africans originated from West-Central Africa [24].

Previous studies have shown that both the mtDNA and autosomal markers (Ancestry Informative Markers; AIMs) indicate a major Amerindian ancestry in Bolivian populations. Most Bolivian mtDNA’s (98.4%) were found to belong to Native American haplogroups. In comparison, analyses of AIMs exhibited an increasingly European admixture (13–25%), while African ancestry in Bolivian populations did not exceed 2% [25,26]. Similar figures were reported in other studies that found almost exclusively Amerindian mtDNA haplogroups [27–31]. Within and between group genetic variability in Bolivian populations is apparently high [27,32]; however, this variability might not apply to all populations within Bolivia [33]. Also in agreement with other studies carried out in Native American populations, the Y-chromosome in Bolivians shows an important introgression of European lineages [21,34].

The first aim of this study was to investigate the European, American, and African ancestry in a Bolivian population that inhabits the Yungas valley. We sampled individuals from both the Nor and the Sud Yungas, the regions where, according to historical documentation, one could expect the greatest impact of the TAST within Bolivia compared to what has been found in previous studies [25]. Second, by means of phylogeographic analysis of selected African mitogenomes, we aimed to obtain greater resolution with regard to their geographic origin in sub-Saharan Africa. That is, we attempted to infer the specific African region(s) from which ancestors of present-day mtDNAs sampled in ‘Afro-Bolivian’ communities might have been brought.

## Material and Methods

### Population samples

We analyzed a total of 105 individuals for their mtDNA hypervariable regions (HVS-I/II) and for a set of 46 AIMs. All individuals were sampled from the department of La Paz, specifically 97 from the province of Nor Yungas and a further eight from the adjacent province, Sud Yungas. Among the Nor Yungas we included 19 individuals originating from the ‘Afro-Bolivian’ community of Tocaña. Samples from Sud and Nor Yungas were merged in a single group for statistical and phylogenetic analyses. Additional analyses were carried out for the samples recruited in Tocaña.

In addition, eight individuals from Tocaña carrying profiles of recent African origin were sequenced for their entire mtDNA genome.

As done in previous studies [25,26], we used 327 samples from the HGCP-CEPH panels [35] as our reference to estimate continental ancestry on the autosomal indels. The reference population panel was as follows: America (*n* = 64), Europe (*n* = 158), and Africa (*n* = 105).

### Ethics statement

Written informed consent was obtained from all sample donors. The institutional review boards of Santiago de Compostela (Spain) approved the present project. The study also abides by the Spanish Law for Biomedical Research (Law14/2007–3 of July).

### Mitochondrial DNA sequencing

The entire control region was amplified using the primers 15997L (5’-CAC CAT TAG CAC CCA AAG CT-3’) and 017H (5’-CCC GTG AGT GGT TAA TAG GGT-3’) for HVS-I and 16555L (5’- CCC ACA CGT TCC CCT TAA AT-3’) and 611H (5’- CAG TGT ATT GCT TTG AGG AGG-3’) for HVS-II.

Each PCR reaction was prepared with 4μl Qiagen 2x Qiagen Multiplex PCR Master Mix, 1μl forward primer, 1μl reverse primer, 2μl DNA (concentration between 0.5–5 ng/μl), and 2μl water. We checked the PCR amplification for success with Polyacrylamide Gel Electrophoresis (PAGE). Subsequently, we used a MultiScreen-PCR 96 Filter Plate (Millipore) to purify all successfully amplified PCR products in a vacuum manifold. For sequencing we used the BigDye Terminator v.3.1. Cycle Sequencing Kit from Applied Biosystems. Each reaction comprised 2μl BigDye Buffer, 0.5μl BigDye Kit, 1μl primer, 5μl PCR product, and 1.5μl water. The sequencing reaction was, like the PCR, conducted either with a Thermal Cycler 2720, and either a GeneAmp PCR System 2700 or 9700 (Applied Biosystems). All sequencing products were purified with ethanol in a 96-Well PCR Plate. We added 1μl 125mM EDTA, 1μl 3M NaOAc, and 25μl ethanol (96%) to each sequencing reaction product. We prepared each sample with 10μl HiDi formamide (Applied Biosystems) for capillary electrophoresis with an ABI 3730xl. Analysis of mtDNA mutations was carried out with the SeqScape v2.1. software.

Sequencing of the whole mtDNA genome followed the same protocol described above. Details on primers for whole mtDNA sequencing were previously published in [36–38].

### Annotation of mtDNA sequences and phylogenetic mtDNA trees

We aligned each sample against the revised Cambridge Reference Sequence (rCRS; [39,40]). All differences from the reference sequence are summarized in S1 Table for the complete control region sequences and in S2 Table for the mitogenomes (GenBank accession numbers from KP635236 to KP635243). A first exploratory haplogroup assignment was performed using Haplogrep [41] and Build 16 of Phylotree [42]. Haplogroup classification was doubled-checked manually. L-haplogroups, unless specified in the text, refer to haplogroups of recent African origin, disregarding macro-haplogroups M and N that are of mainly non-Sub-Saharan origin; i.e. L(×M,N). We followed the phylogenetic approach to scan the sequences as an *a posteriori* sequence quality control [43,44]; this filter was also applied to the data collected from the literature.

We initially built phylogenetic trees using mtPhyl v3.520 (http://eltsov.org). However, as the software is not based on the most recent mitochondrial phylogeny, phylogenetic trees were adjusted manually according to mtDNA tree Build 16 of Phylotree (http://www.phylotree.org) and the phylogeny was updated according to the new findings in the present study.

In order to determine the most likely (phylo)geographic origin of the eight mitogenomes from Tocaña, we analyzed our control region data together with a database that comprised 2,235 mitogenomes belonging to haplogroup L(×M,N) sampled mainly in Africa (S3 Table; S1 Fig). Eventually we focused on 152 L-mitogenomes (S4 Table) that formed a sub-branch, together with the genomes from Tocaña generated in the present study. Out of the 152 samples, we used 101 samples from previously published studies or GenBank. The remaining 51 samples were downloaded from The 1000 Genome Project (http://www.1000genomes.org/) using the same procedure as in [45].

### AIM-INDEL genotyping

All individuals were genotyped for 46 AIMs [46] using a multiplex PCR amplification, as done in previous studies [25,26]. In brief, each PCR reaction was a mixture of 5μl 2× Qiagen Multiplex PCR Master Mix, 1μl 10× Primer Mix, 0.5μl DNA (concentration between 0.5-5ng/μl), and 3.5μl water. The samples were prepared for capillary electrophoresis by adding 11.5μl Hi-Di Formamide (Applied Biosystems) and 0.3μl Liz-500 Size Standard (Applied Biosystems) to 0.8μl PCR product. A 3130*xl* Genetic Analyzer (Applied Biosystems) was used to separate DNA fragments by size. Analysis of indels was conducted by applying the software GeneMapper (Applied Biosystems). The genotyping results are reported in S5 Table.

### Mitochondrial DNA and autosomal data analyses

Diversity indices computed on mtDNA sequences were obtained using DnaSP v.5. [47]. Genetic ancestry analysis based on indel data (AIMs) was carried out with ADMIXTURE software [48], which uses a maximum likelihood estimation of individual ancestries from multilocus genotypes. This software was used to estimate percentages of admixture in the Yungas to a continental level considering three main continental groups (sub-Saharan Africa, Native Americans, and Europeans) represented by 327 samples from the Human Genome Diversity Cell Line Panel (HGDP-CEPH). Note that ancestry inferences for individuals have to be considered with care owing to the variability of estimates when using a limited number of AIMs [49], but inferences based on population samples can be very robust, even when using a limited number of individuals [26,49].

For Multidimensional Scaling (MDS) we used the software PLINK 1.07 [50] and R v.3.0.2 (http://www.r-project.org). We first created an allele-sharing genetic distance matrix to calculate the Identical by State (IBS) values between pairs of individuals.

Admixture analysis was additionally carried out on Bolivian L-mtDNA haplotypes (‘Afro-Bolivian’) in order to allocate the variability observed in the most likely source population in Africa. An admixture analysis based on haplogroup frequencies, as performed in [15], was not possible given the limited number of Bolivian L-mtDNAs, which does not allow realistic inferences of frequency estimates. Therefore, we carried out an analysis based on haplotype sharing [51], which is an extension of previous proposals [7,52], by allowing step-mutation differences between ‘Afro-Bolivian’ haplotypes and the source populations. A database of >13,700 African profiles was used and six different sub-continental source regions were considered: North (*n* = 3,830), West-Central (*n* = 6,868), Southwest (*n* = 522), South (*n* = 357); Southeast (*n* = 1,118), and East (*n* = 1,010) Africa (S6 Table).

### Y-chromosome data

A few individuals analyzed in the present study had been previously analyzed for Y-chromosome STR and SNP markers [21]. Here we used the haplogroup classification of these lineages for comparison purposes, and derived some population inferences not made before regarding gender biases in the ‘Afro-Bolivian’ community. The relevant data used in the present study were incorporated into S1 Table.

## Results

### Molecular diversity of mtDNAs in the Yungas

On average, haplotype diversity is higher in the rest of Bolivia (excluding the Yungas) than in the Yungas [25] probably reflecting the more isolated character of the Yungas valley (Table 1). Within the Yungas, the Tocaña seems to contribute more to the haplotype diversity than the remaining population of this region.

**Table 1:** Sequence diversity indices in Bolivian mtDNAs. The indices were computed using the common segment of the HVS-I region from position 16090 to 16365.

Population groups	*n*	*k*	*S*	*H*	*π*	M
Bolivians (without Yungas)*	720	306	134	0.976±0.002	0.02237±0.00041	6.130
Yungas	105	52	59	0.943±0.016	0.02364±0.00168	6.500
Yungas without Tocaña	86	44	54	0.920±0.024	0.01895±0.00161	5.212
Tocaña	19	11	26	0.924±0.037	0.02990±0.00230	8.222

NOTE: *n* = sample size; *k* = number of haplotypes; *S* = number of segregating sites; *H* = haplotype diversity; *π* = nucleotide diversity; *M* = average number of pairwise differences.

* Data from [25].

However, nucleotide diversity is highest in Tocaña, reflecting the larger contribution of mtDNAs of recent African origin to their pool. The nucleotide diversity decreases substantially in the Yungas when the Tocaña are excluded. The average number of nucleotide differences shows the same pattern as this measure is directly correlated with nucleotide diversity.

### Phylogeography of control region mtDNA haplotypes in the Yungas

Aproximately 81% of the mtDNA haplotypes observed in the Yungas are of Native American origin, and virtually all of the remaining ones belong to typical haplotypes of recent sub-Saharan origin.

**Fig 1:**
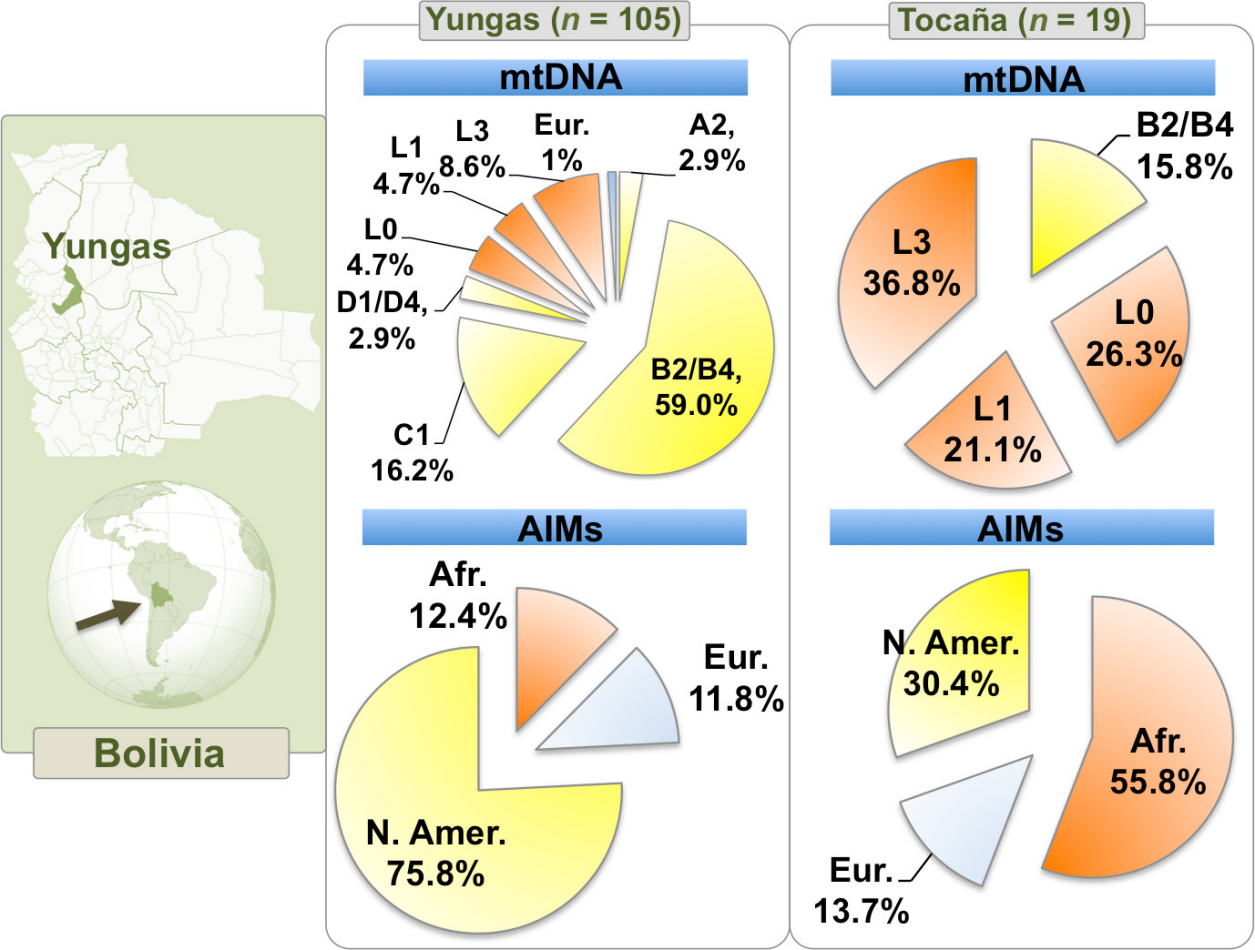
Map showing the location of the Yungas region within Bolivia. The pie charts indicate the proportions of continental ancestries on the mtDNA and biparental (AIMs) markers; exact values for continental ancestry based on AIMs are shown in S7 Table.

Native American haplotypes in the Yungas belong to one of the typical haplogroups commonly found in the Americas [53–55], the most numerous haplogroup being B2/B4 (59%), followed by C1 (16.2%), A2 (2.9%) and D1/D4 (2.9%) (Fig 1). The D4 haplotype belonged specifically to haplogroup D4h3a, a clade that is thought to have arrived to South America along the Pacific coast [54]. Only one haplotype in the Nor Yungas seems to be of European origin (haplogroup HV0).

The most common Native American motif in the Yungas is that represented by the diagnostic HVS-I motif of B4 (T16189C-T16217C) with the C16188T transition on top (18% of the total haplotypes in the Yungas). This motif is very common in Bolivia [25,26,32,56] and is also present in other Andean locations: Uros (Peru) [57], Jujuy (Argentina) [58], and Coyas (Argentina) [58,59]. Given that the C16188T transition is mutationally stable (only nine hits in Phylotree and 10 in Soares et al. [60]), this motif probably represents a lineage (haplogroup) that has evolved locally in the Andean region. Its spatial distribution could have originated from historical trans-Andean movements.

About 18% of the haplotypes found in the Yungas are of recent sub-Saharan ancestry, mainly represented by different L-haplogroups: L0 (4.7% of the total Yungas), L1 (4.7%), and L3 (8.6%) (Fig 1). The great majority of the African lineages were observed in the locality of Tocaña (16 out of 19 L-haplotypes; 84% of all Tocañas). The most common L-haplogroup in the Yungas is L1c3b1a, which makes-up 26.3% of all L-haplotypes.

Singleton haplotypes are less common in the Yungas when compared to other Bolivian regions. Thus, many haplotypes appear at least twice in this region, and this affects not only Native American haplotypes but also those of recent African ancestry. This fact explains the reduced haplotype diversity existing in the region when compared to the rest of the country, adding support to the idea that the Yungas are a relatively isolated population within Bolivia.

### Phylogenetic trees of ‘Afro-Bolivian’ mitogenomes

**Fig 2:**
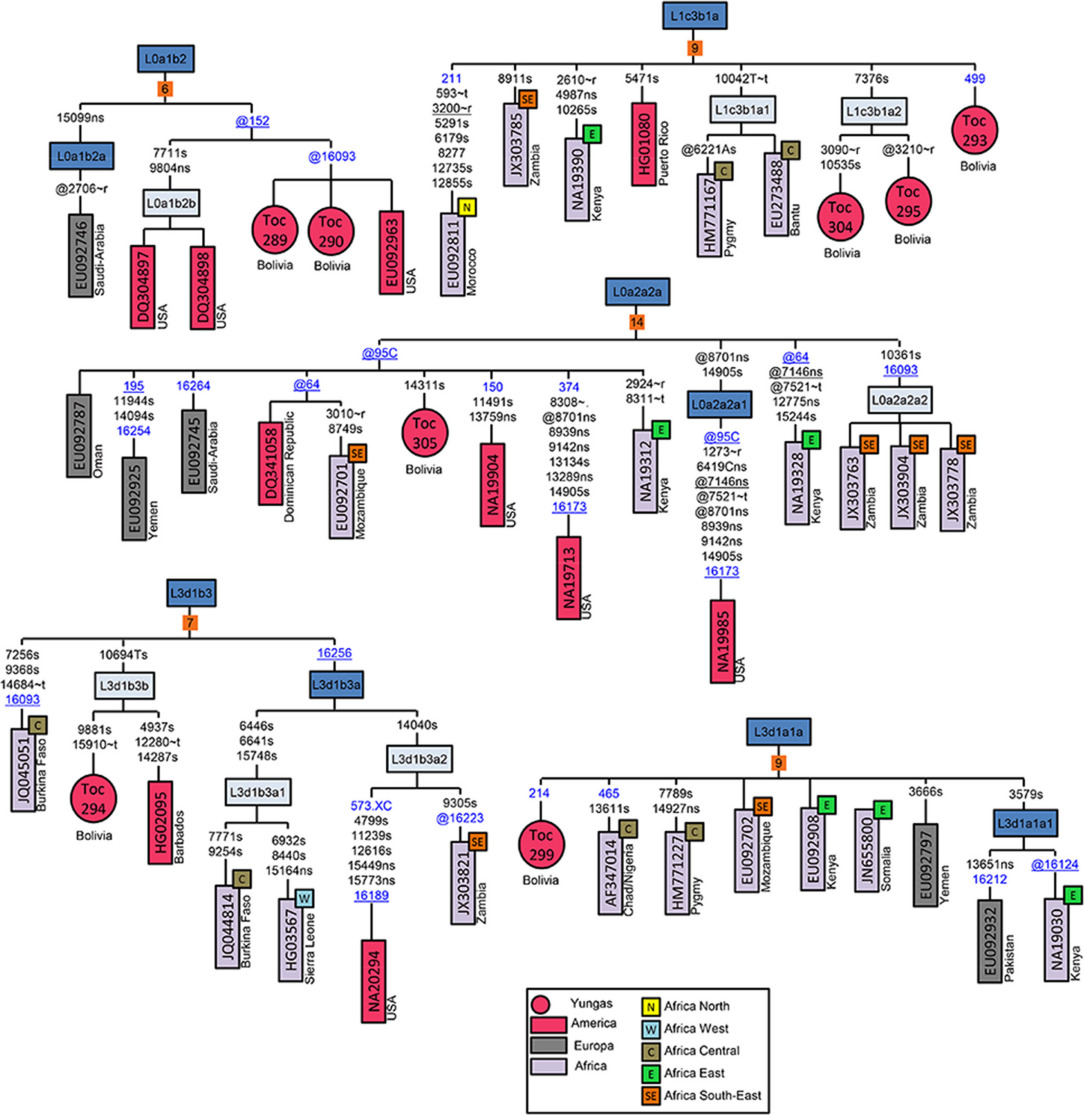
Maximum parsimony tree of Yungas mitogenomes. Variant calling is referred to the rCRS [39]. Mutational changes are shown along branches (those falling in the control region are in blue); mutations are transitions unless a suffix A, C, G, or T indicates a transversion. Other suffixes are: insertions (.X), synonymous substitutions (s), non-synonymous substitutions (ns), mutational changes occurring at tRNAs (~t), and mutational changes occurring at rRNAs (~r). A back mutation is represented with the prefix “@”, whereas an underlined mutation represents a recurrent mutation in the phylogeny shown in the figure. As per common practice, variants at positions 16182 and 16183, variation around position 310, and length or point heteroplasmies were not considered for phylogenetic reconstruction. The mitogenomes analyzed in the present study are indicated as red circles. Numbers in the orange squares attached to haplogroup labels indicate the number of mitogenomes within haplogroups.

Mitogenomes were obtained from eight individuals from the locality of Tocaña whose control region revealed they belonged to some L-(sub)haplogroup. Complete genome sequences allowed their haplogroup assignation to be refined as follows: #Toc289 and #Toc290 belonged to haplogroup L0a1b2, #Toc305 to L0a2a2a, #Toc293 to L1c3b1a, #Toc295, and #Toc304 to (the newly defined) haplogroup L1c3b1a2, #Toc299 to L3d1a1a, and #Toc294 to (the new sub-branch) L3d1b3b (Fig 2). Phylogenetic tree analyses suggest that the geographic origin of all these mitogenomes cannot be easily traced back to a single geographic region in Africa, although some tentative geographic inferences can be made.

Samples #Toc289 and #Toc290 share haplogroup L0a1b2 status with three individuals from the USA and one individual from Saudi-Arabia (that could be further ascribed to haplogroup L0a1b2a; Fig 2). The two samples from Tocaña, together with another mitogenome from the USA (EU092963), form a sub-branch based on @152 and subsequently on @16093; both are highly mutationally unstable variants [60]. There are two other individuals from the USA who share the @152 reversion (but not @16093); however, they also have the variants T7711C and G9804A. The phylogeographic characteristics of L0a1b (that is, the most immediate ancestral node of L0a1b2) do not help allocate its members to specific regions in Africa; but the L0a1b branch clearly shows that this branch is well represented in America (37% of the haplotypes; S2 Fig). Haplotypes at the ancestral node L0a1 show that some branches are well represented in East and South Africa, while others are found in North Africa (Egypt and Morocco) and also in Chad.

Individual #Toc305 matches haplogroup L0a2a2a within a sub-branch that is determined by the @95C reversion together with two samples from the USA, one from the Dominican Republic (overall the ‘Afro-American’ component accounts for 44% of this clade), three from the Near East (Oman, Yemen, Saudi-Arabia), and one each from Kenya and Mozambique (Fig 2). The neighboring L0a2a2a2 sub-branch (based on mutations T10361C and T16093C) comprises three mitogenomes from Southeast Africa (Zambia); the sub-branch L0a2a2a1 (based on @A8701G and G14905A) includes one from the USA. One individual from Kenya could not be further classified within L0a2a2a. On the whole, the sub-continental African region that is best represented in L0a2a is the Southeast and East, followed by the Middle East (S2 Fig).

The three ‘Afro-Bolivians’ #Toc293, #Toc295, and #Toc304 belong to L1c3b1a. This sub-haplogroup is also represented by one mitogenome each from Kenya, Zambia, a Bantu-speaking Fang, Pygmy, and Morocco. Apart from the Afro-Bolivian mitogenomes, this sub-branch comprises only one additional sample from the Americas, namely, from Puerto Rico; ‘Afro-Americans’ represent 44% of L1c3b1a. Other members of L1c3b1 comprise one haplotype sampled in Colombia and one from the Western Sahara, but also one each from Mozambique and Nigeria (Yoruba) belonging to sub-branch L1c2b1b (S3 Fig).

The Tocaña #Toc299 belongs to haplogroup L3d1a1a, a clade that clusters mitogenomes sampled in Kenya, Mozambique, Nigeria/Chad (Yoruba), Pygmy, Yemen, and Somalia. The sub-haplogroup L3d1a1a1 comprises one individual from Kenya and one from Pakistan. Other members of L3d1a1 fall in East (Somalia) and South Africa; but also into West and Central Africa (Gambia, Nigeria, etc) (S4 Fig).

Individual #Toc294 can be allocated to haplogroup L3d1b3 together with one individual from Barbados, one from Burkina Faso, and the sub-clade L3d1b3a (which is represented by one individual each from Burkina Faso, Sierra Leone, Zambia, and USA). Within other branches of L3d1b, the African region that is most represented is West-Central Africa (S4 Fig).

Finally, some phylogeographic connection can also be established between the mitogenomes analyzed in the present study and other American locations (S2 Text).

### Admixture analysis of ‘Afro-Bolivian’ mtDNA control region haplotypes

A total of 19 mtDNAs (10 different HVS-I haplotypes) belonging to haplogroup L were observed in Yungas. An admixture analysis was carried out with these L-Bolivian haplotypes in order to estimate admixture proportions from different sub-continental African regions (Table 2).

**Table 2:** Admixture proportions, *P*_0_, *P*_1_, and *P*_2_ (and 95% C.I) of ‘Afro-Bolivian’ mtDNA haplotypes regarding their main sources in sub-continental regions in Africa.

African region	*P*_0_	95% C.I. (*P*_0_)	*P*_1_	95% C.I. (*P*_1_)	*P*_2_	95% C.I. (*P*_2_)
North	0.0189	0.0180–0.0198	0.0344	0.0332–0.0356	0.0514	0.0499–0.0528
West-Central	0.1238	0.1217–0.1260	0.1521	0.1498–0.1545	0.2045	0.2019–0.2071
Southwest	0.5063	0.5030–0.5095	0.3617	0.3586–0.3649	0.3314	0.3283–0.3345
South	0.0054	0.0049–0.0059	0.0859	0.0841–0.0877	0.0986	0.0966–0.1005
Southeast	0.2607	0.2579–0.2636	0.2544	0.2515–0.2572	0.2058	0.2032–0.2085
East	0.0849	0.0831–0.0867	0.1114	0.1094–0.1135	0.1083	0.1063–0.1104

In contrast with what is generally observed in other American locations (including Brazil and Colombia), where most of the ‘Afro-American’ variation can be allocated to West-Central Africa [8,14–16,18,20], ‘Afro-Bolivian’ mtDNAs are mainly allocated to Southwest (mainly represented by Angola and Cabinda), and Southeast Africa (mainly represented by Mozambique). The contributions of these African regions differ depending on the proxy considered; when the inference is based on exact matches (*P*_0_) the Southwest contributes 51% of the variation in ‘Afro-Bolivians’ compared to 26% from the Southeast, while the proportions are, respectively, 36% and 25% if we consider one mutation-step difference between haplotypes (*P*_1_) (Table 2). West-Central Africa is the third main contributor to ‘Afro-Bolivians’. Interestingly, according to this analysis, East Africa would contribute between 8% and 11% of the African component in these ‘Afro-Bolivians’.

### Multidimensional Scaling based on AIMs

**Fig 3:**
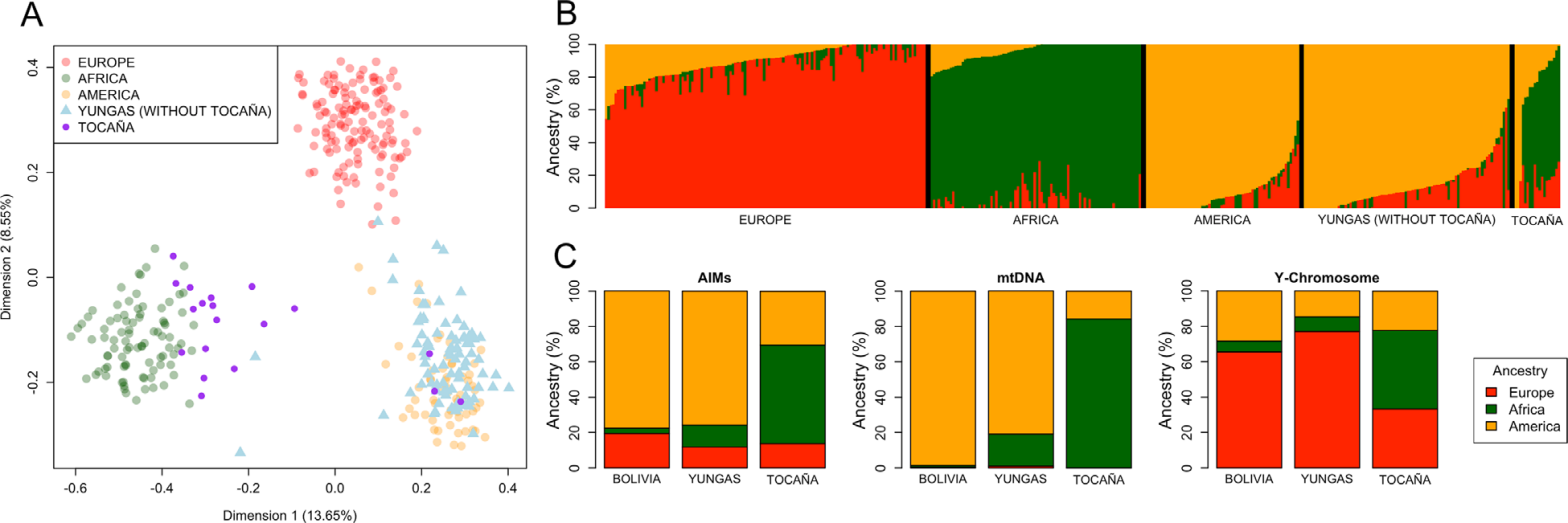
Multidimensional Scaling (A), and admixture analysis based on AIMs (B) of the Yungas population samples. Partitioning of continental ancestry in Yungas compared to other Bolivian populations as estimated using AIMs, mtDNA, and Y-chromosome data (C). The label “Bolivia” in (C) indicates ancestry estimates recomputed using the autosomal data generated by Taboada-Echalar et al. [25] (their Bolivians did not contain samples from the Yungas Valley) and considering the same computational procedures and reference population datasets used in the present study. In addition, Y-chromosome estimates in (C) were inferred from the data in Cárdenas et al. [21]

The MDS plot of Fig 3A shows the three main ancestral components (Europe, Africa and Native America) occupying the vertices of a triangle. Dimension 1 accounts for 14% of the variation and separates mainly Africans from the rest, while Dimension 2 accounts for 9% of the variation and separates Europeans from the rest. Most of the individuals from the Yungas are proximal to the Native American pole of the plot. Only a few of them show a projection to the European cluster. On the other hand, the plot clearly shows the affiliation of most individuals from Tocaña (and two other individuals from the Nor Yungas) to the African cluster; only 3 out of all 19 Tocaña samples have a very low African membership, ranging from 0.8% to 1.2% (see below), and these are the Tocaña individuals who fall within the Native American cluster (Fig 3A).

### Ancestry analysis based on AIMs

Individual ancestry, as inferred from autosomal markers, is represented in the ADMIXTURE barplots of Fig 3B. The partitioning of continental ancestry in the Yungas is as follows: 76% Native American, 12% European, and 12% African (S7 Table). In contrast, the average African ancestry for the 19 individuals from the community of Tocaña is as high as 56%, while the Native American ancestry falls to 30% and the European component to 14% (S7 Table).

The maximum value for African ancestry in the Yungas could be attributed to one individual from Tocaña showing 78% African ancestry (S7 Table). The maximum value of European ancestry was 62%; however, among the Tocaña, the maximum value was just 28%.

In contrast, the Native American genetic component in the Yungas was highly variable, ranging from 1% and 100% (S7 Table).

### Gender bias in the Yungas

The existence of a strong gender bias in the gene pool of Bolivia has been previously reported [21]. While the mtDNA in the country is mainly of Native American ancestry (98.4%) [25], the Y-chromosome shows a major introgression of European ancestry (65%) [21]. Autosomal data indicates also a major Native American ancestry (71%) compared to a lower European one (25%) [25]. The African ancestry in the Bolivians analyzed in the literature is very low for uni- and bi-parental markers [25], ranging from <1% on the mtDNA and the autosomal markers to 6% on the Y-chromosome (Fig 3C).

The scenario observed in the Yungas valley is different to that in the rest of Bolivia (Fig 3C). Although there is also a marked gender bias, the main distinctive feature of the Yungas with respect to the rest of Bolivia is the introgression of African ancestry in the gene pool of the region. Thus, the African ancestry on the autosomes, the mtDNA and the Y-chromosome is, respectively, 12%, 18%, and 8%. In particular, the locality of Tocaña concentrates the main African ancestry in the Yungas (Fig 3C). The partitioning of ancestries in this locality for the autosomes, the mtDNA, and the Y-chromosome, respectively, is as follows: (i) African ancestry: 56%, 84%, and 44%; (ii) Native American ancestry: 30%, 16%, and 22%; and (iii) European ancestry: 14%, 0%, and 33%.

## Discussion

Enslaved Africans who embarked in Senegambia and arrived in Upper Peru principally entered the Americas *via* Cartagena de Indias (Colombia), whereas those embarked from West-Central Africa mainly had to first pass Río de la Plata (present day Uruguay / Argentina). African enslaved people arriving at Río de la Plata, predominantly from Angola, either had to remain in the region where they disembarked (e.g. Uruguay) or were further transshipped to Upper or Lower Peru, but also to Chile [13]. As Upper Peru had no immediate access to the sea, other principal ports of arrival were Recife, Salvador-Bahía and Río de Janeiro in Brazil, and Panamá and El Callao in Peru [4,11].

The results of the present study indicate that the Yungas have a characteristic genetic composition when compared to other Bolivian regions owing to the impact of the TAST in the region coupled with its geographic isolation. The locality of Tocaña contributes most significantly to this distinctiveness, given that most of the ‘Afro-Bolivian’ (mtDNA and Y-chromosome) haplotypes observed in the country were found here. Furthermore, autosomal African ancestry is significantly higher in the Yungas than in other parts of the country, a feature that can also be mainly attributed to the Nor Yungas community of Tocaña.

Mitogenomes observed in ‘Afro-Bolivian’ communities cannot be traced back to one single geographic location in Africa. A West or West-Central genetic impact on ‘Afro-Bolivian’ communities cannot be excluded. For instance, the ‘Afro-Bolivian’ individuals #Toc293, #Toc295, and #Toc304 form the sub-branch L1c3b1a, including one Pygmy and one Bantu individual. Sub-branch L3d1a1a with #Toc299 also comprises one individual from Nigeria/Chad (Yoruba), plus one Pygmy. Sub-branch L3d1b3, with #Toc294, includes two individuals from Burkina Faso and one from Sierra Leone.

Contrary to expectations [14], no mitogenome of the database from either Angola or Congo could be found in the same branches as the eight individuals from Tocaña analyzed. Neither could we found a direct connection between modern ‘Afro-Bolivians’ and Senegambia, as no individual from Senegal or Gambia (or from nearby countries such as Guinea-Bissau and Mauritania) was affiliated to the same sub-branches as the samples from Tocaña. Only one individual from Sierra Leone belonged to L3d1b3 (like #Toc294). Instead, our data indicate a genetic impact on ‘Afro-Bolivians’ from East or Southeast Africa, but surprisingly also from the Middle East. Individual #Toc305 belongs to the same haplogroup (L0a2a2a) as three individuals from Oman, Yemen, and Saudi-Arabia, together with one individual from Mozambique, three from Zambia, and two from Kenya. Likewise, ‘Afro-Bolivian’ #Toc299 can be found in the same haplogroup (L3d1a1a) as two samples from Kenya and one from Somalia, and one sample from the Middle East, namely from Yemen. Individuals #Toc293, #Toc295, and #Toc304 also form a sub-branch (L1c3b1a) together with one sample from Kenya and one from Zambia.

Analyses of admixture based on a large dataset of African mtDNA control region sequences are very strongly consistent with these phylogeographic inferences. Thus, although these analyses indicate that the main contribution to ‘Afro-Bolivians’ comes from the West-Central and Southwest Africa (together >50–60%), a significant proportion of African ancestry comes from Southeast (>20%) and East Africa (>8%).

The Y-chromosome also shows signatures of a Southeast African component in Bolivia. By searching the minimum Y-STR haplotype in the Y-chromosome Reference Database (YHRD; http://yhrd.org) of those profiles from Bolivia classified as belonging to a Y-chromosome haplogroup [21], we observed that a few of them (e.g. #Tocaña291) have a clear geographic prevalence in Southeast Africa (S3 Text).

Contrary to expectations, the results on the mtDNA from the Yungas can only be understood if we assume an important contribution of Southeastern Africa (and possibly East Africa) in the Yungas. It is known that Southeastern Africa became an increasingly important slave provider towards the end of the TAST. According to the TAST database, Southeast Africa (and Indian Ocean islands) started to provide enslaved people between 1661 and 1665. This region was the second largest slave supplier (after West-Central Africa) between 1816 and 1820, after the British abolished the slave trade in their English colonies in 1808 [1,10]. Most of the enslaved people in Southeastern Africa, as many as 112,800 individuals, embarked between 1821 and 1830. Their principal destination was Brazil (70.6%), followed by the Caribbean (19.9%) [1]. The British abolished the slave trade in its colonies in 1808, and forced other European countries to follow suit. However, their efforts became noticeable on the Atlantic coast only after 1840 and even later in East Africa [10]. The East African slave trade was even more complex than its Western and West-Central African counterparts. Slaves in East Africa were not only destined for the TAST, but also for the Trans-Saharan trade and for the trade across the Red Sea and Indian Ocean [61]. Most enslaved Africans who left East / Southeastern Africa for the Americas embarked on ships in Mozambique; trade in this region was particularly active between 1836 and 1840 involving around 35,000 slaves [1].

Concurrently, in Bolivia, emancipation from the Spanish colonists in 1825 did not completely abolish slavery in the country, in spite of several attempts. Slavery was effectively abolished only after the agrarian reform initiated in 1952 [2,11]. Therefore, given the late end of the TAST in Southeastern Africa and the equally late abolition of slavery in Bolivia, some enslaved Africans might have continued to arrive in the country towards the end of the TAST. Thus, a genetic impact from East and Southeastern Africa on ‘Afro-Bolivians' would be reasonable. At the end of the TAST most enslaved people were forced to move to Brazil, Cuba and Puerto Rico [10]. As Bolivia had no immediate access to the sea, the main ports of arrival for slaves that reached Bolivia included those in Brazil such as Recife, Salvador-Bahía and Río de Janeiro [3, 7]. Thus, enslaved Africans who reached Bolivia towards the end of the TAST might have disembarked in Brazil.

The reason for a genetic input from Near Eastern countries such as Saudi-Arabia, Yemen, and Oman in ‘Afro-Bolivians’ is rather unclear. It might indirectly reflect a connection between (i) the TAST and the trans-Saharan Slave Trade, and/or (ii) the TAST and the slave trade along the East African coast to Southeast Africa (Mozambique) (see S4 Text). The Bight of Benin, the Gold Coast, the Bight of Biafra, and Senegambia were the regions with the strongest connections to the Arab slave trade, whereas the trans-Saharan slave trade and the TAST overlapped predominantly in Senegambia. In contrast, the Islamic influence on the slave trade was minimal at the Bight of Biafra and literally absent in West-Central Africa. However, reportedly up to 40% of all slaves who were forced to leave Africa between 1500 and 1800 can be attributed to the Arab slave trade [61]. In any case, based on the genetic and historical information currently available, we cannot make conclusive arguments about a possible a connection between the Arab slave trade and ‘Afro-Bolivian’ communities.

Finally, the present results are also important for revealing different ancestral continental components in the Yungas that are different from those in Bolivia (Fig 3), as well as the existence of a strong gender bias in the region. Understanding patterns of admixture and population sub-structure can be relevant in other fields of biomedical research. For example, an elevated European ancestry in ‘US Latinas’ on chromosome regions 6q25 and 11p15 increases their risk of suffering from breast cancer [62]. Overall, the results of the present study reflect the existence of a large effective population size of European colonizers, represented mainly by males (in the Yungas, 77% of the Y-chromosome haplotypes are of European origin *vs*. 19% of the mtDNA haplotypes). The Native American male population suffered the consequences of the conquest more dramatically, hence the Native American component observed in today’s Yungas population could be mainly preserved in Bolivian females. The African ancestry is mainly concentrated in the Nor Yungas locality of Tocaña, and is particularly high on the mtDNA (84%). The geographic isolation of the Yungas valley may have contributed to better preserving the African character of the region and the Native American component after admixture with local natives. It is also noteworthy that the data tentatively suggest that the TAST contributed more African females than males to the Yungas, or alternatively that the mortality rate was higher in males than in females. This inference is based on the fact that the African component is significantly higher in the mtDNA [84%] than in the Y-chromosomes [44%], although part of this signal could have been blurred by the important genetic introgression of European Y-chromosomes in the Yungas. Another possible explanation is that gender bias affected ‘Afro-Bolivians’, as much as it affected Native Americans.

## Conclusions

The present study has shown that the African origin of individual ‘Afro-Bolivian’ mitogenomes cannot be easily resolved using mtDNA data. As previously advised [14,19], caution is warranted when dealing with claims made by testing companies aimed at tracing the ancestry of a particular customer using uniparental markers. This, however, does not preclude making overall demographic (population) inferences by considering the ‘Afro-Bolivian’ sample. Unexpectedly, the results show not only a rather important genetic impact from East and Southeast Africa, but also from the Middle East. Given that 40% of all enslaved people who were forced to leave Africa between 1500 and 1800 can be linked to the Arab slave trade [61], both the TAST and the Arab slave trade within Africa might have overlapped to a great extent, one that is now being indirectly reflected in ‘Afro-Bolivians’.

## Supporting Information

S1 Fig Map showing the location, sample size and origin in Africa of the global database of African mitogenomes (S3 Table). (TIF)

S2 Fig Phylogenetic analysis of L0a mitogenomes related to those observed in the Yungas mitogenomes. More details are given in legend of Fig 2.
(PDF)

S3 Fig Phylogenetic analysis of L1c3 mitogenomes related to those observed in the Yungas mitogenomes. More details are given in legend of Fig 2.
(PDF)

S4 Fig Phylogenetic analysis of L3d1 mitogenomes related to the ones observed in the Yungas mitogenomes. More details are given in legend of Fig 2.
(PDF)

S1 Table Mitochondrial DNA control region haplotypes from the Yungas and their haplogroup classification. Y-chromosome haplogroup data derived from Cárdenas et al. [21] were also added.
(XLSX)

S2 Table Complete genomes analyzed in the present study. (XLSX)

S3 Table References to the global database of African mitogenomes. (XLSX)

S4 Table Selection of mitogenomes used for the trees in S2–S4 Figs. (XLSX)

S5 Table Genotype data of the AIMs obtained in the Yungas samples. (XLSX)

S6 Table Origin of the African samples used in the present study for admixture analysis of mtDNA control region profiles. (XLS)

S7 Table Continental ancestry in the Nor and Sud Yungas as inferred from AIMs. (XLSX)

S1 Text Notes on the TAST. (DOC)

S2 Text Phylogeographic connections between Afro-Bolivian mtDNAs and other American locations. (DOC)

S3 Text Frequency maps showing the result of searching Bolivian minimum Y-chromosome haplotypes of African ancestry in the Y-chromosome Reference Database (YHRD; http://yhrd.org). (DOCX)

S4 Text Arab slave trade and its connections with the TAST. (DOCX)
