# Supplementary figures and images for: The Genomic Legacy of the Transatlantic Slave Trade in the Yungas Valley of Bolivia
Source: PLoS One. 2015 Aug 11;10(8):e0134129. doi: 10.1371/journal.pone.0134129 (PMC4532489; doi:10.1371/journal.pone.0134129)

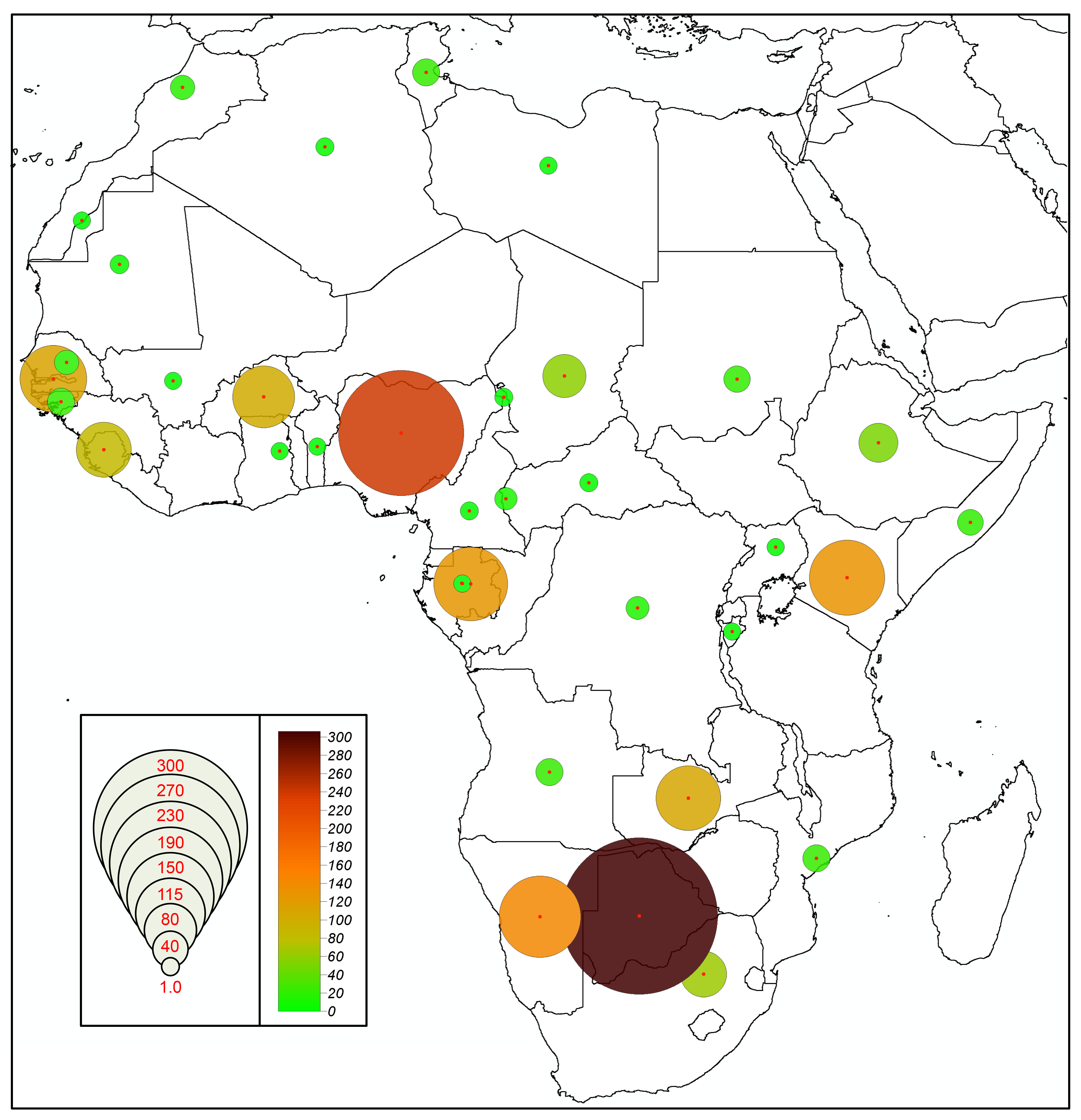

Supplement: S1 Fig — (TIF) [file pone.0134129.s001.tif]

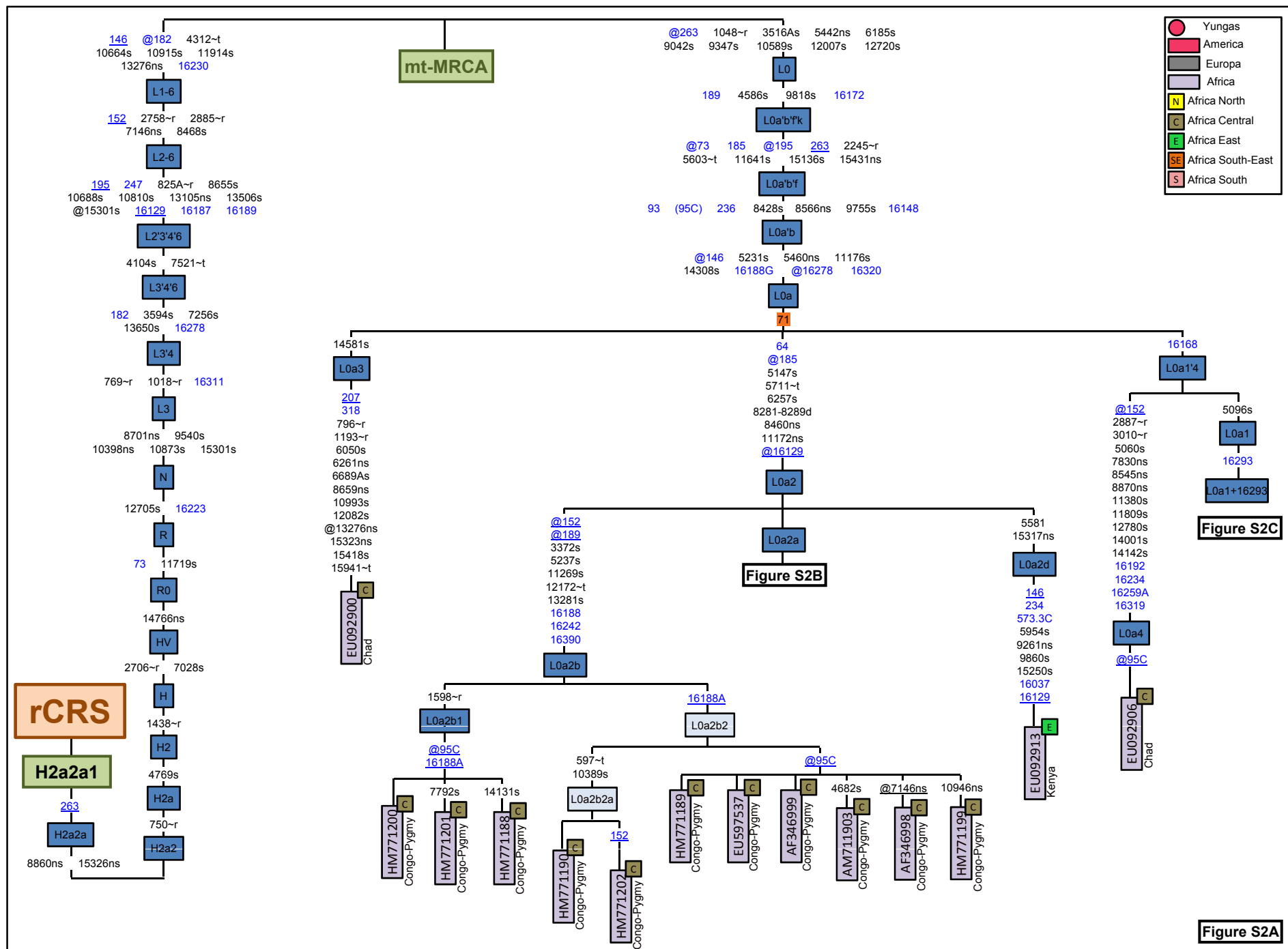

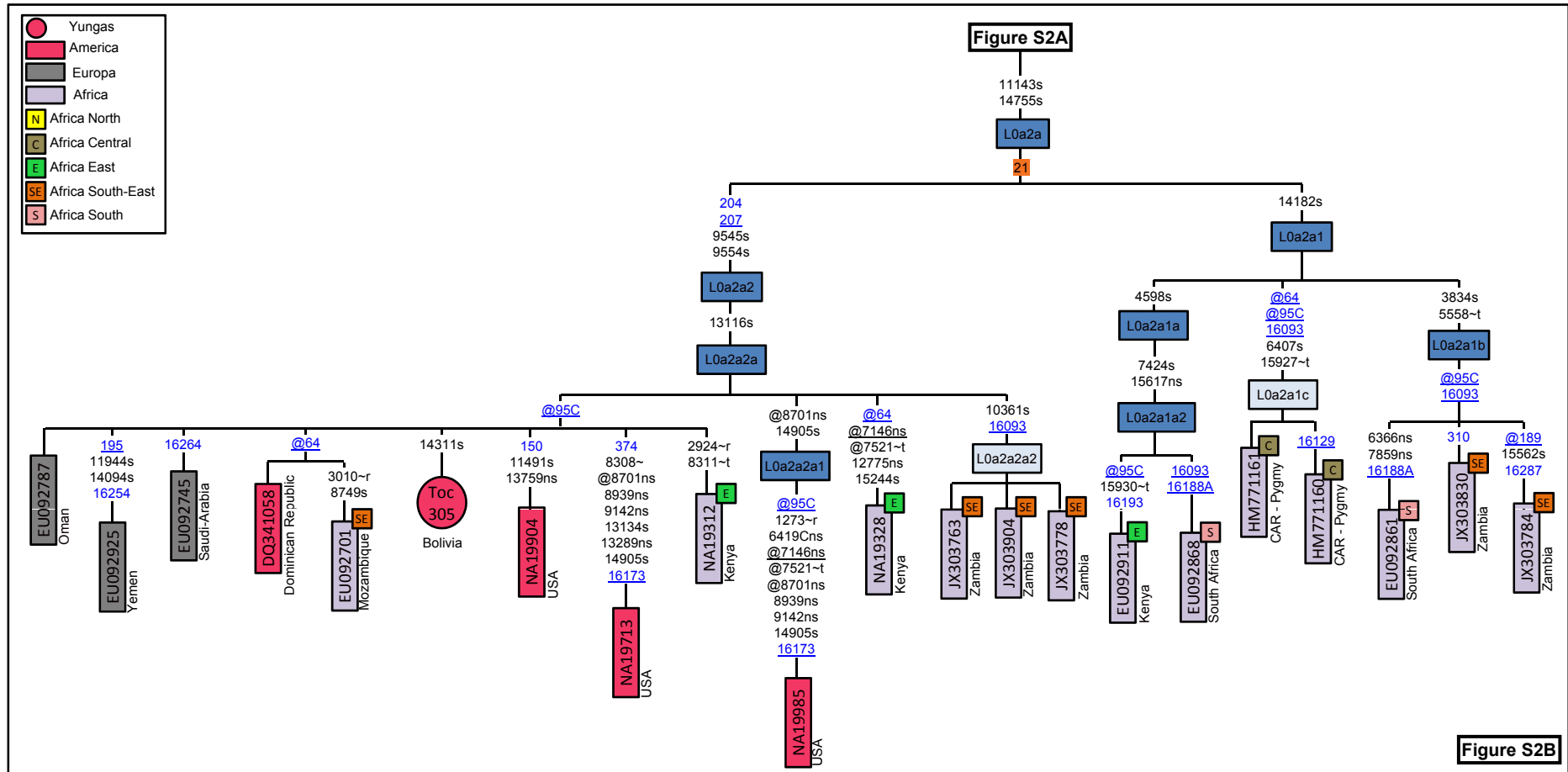

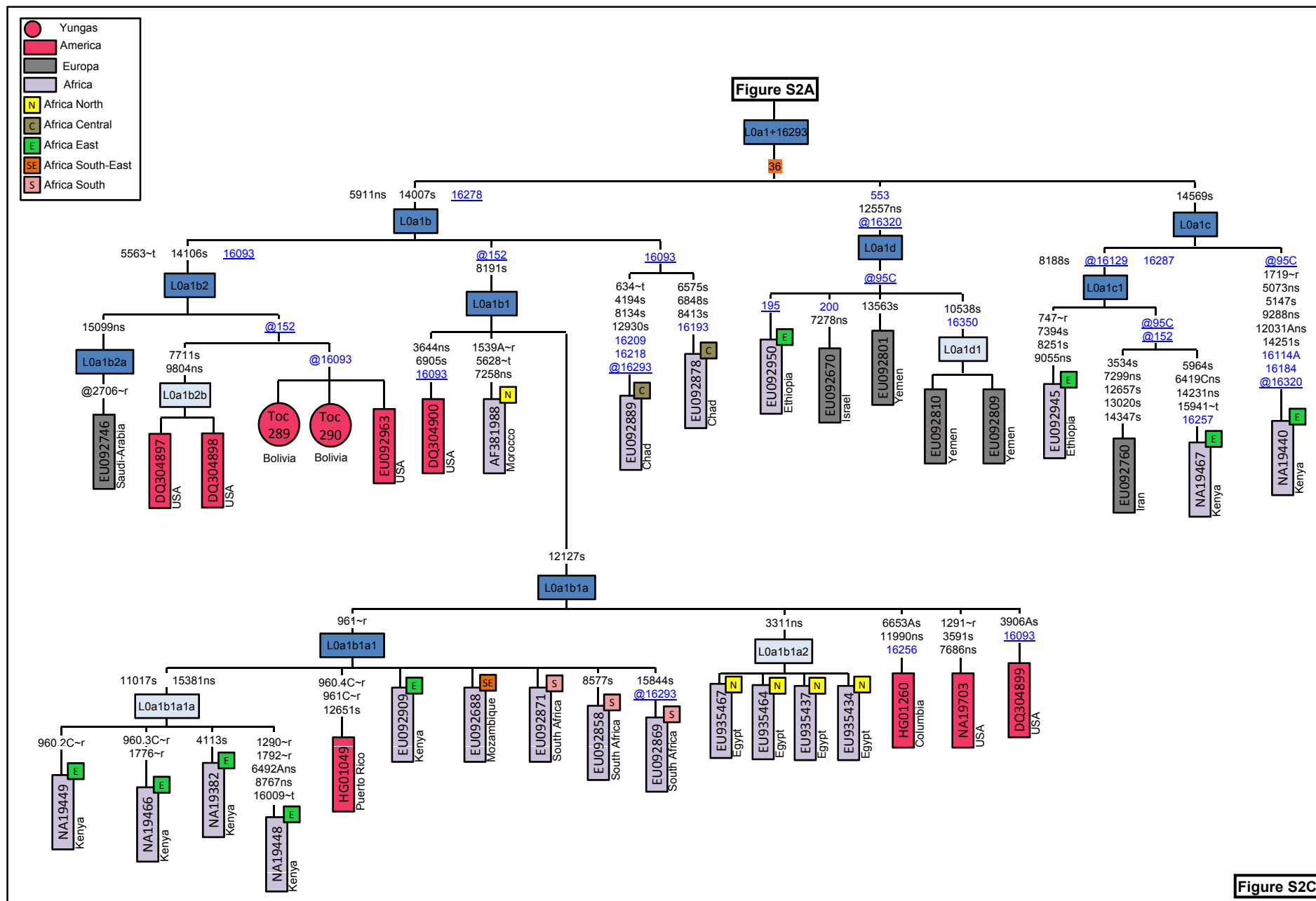

Supplement: S2 Fig — More details are given in legend of Fig 2. (PDF) [file pone.0134129.s002.pdf]

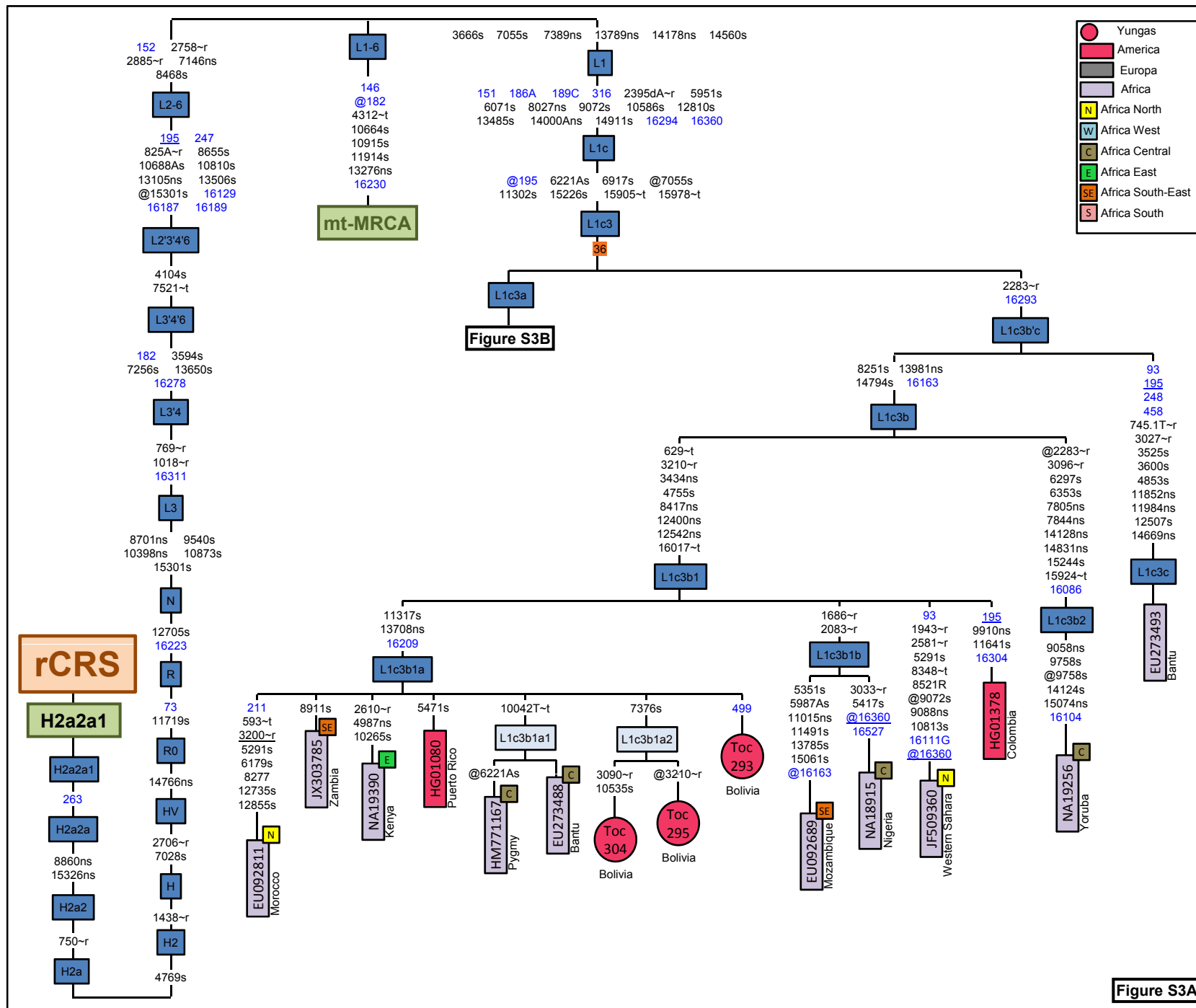

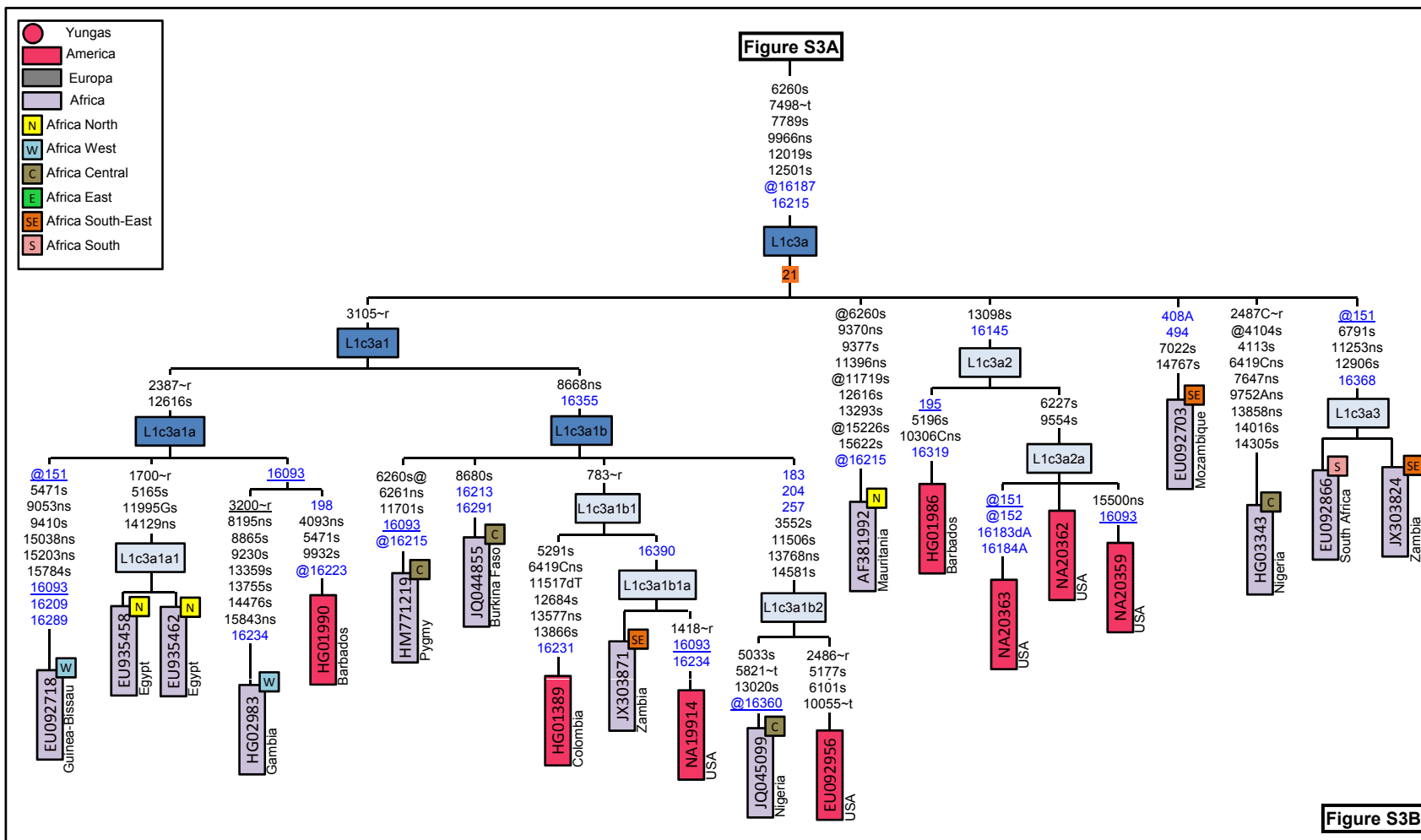

Supplement: S3 Fig — More details are given in legend of Fig 2. (PDF) [file pone.0134129.s003.pdf]

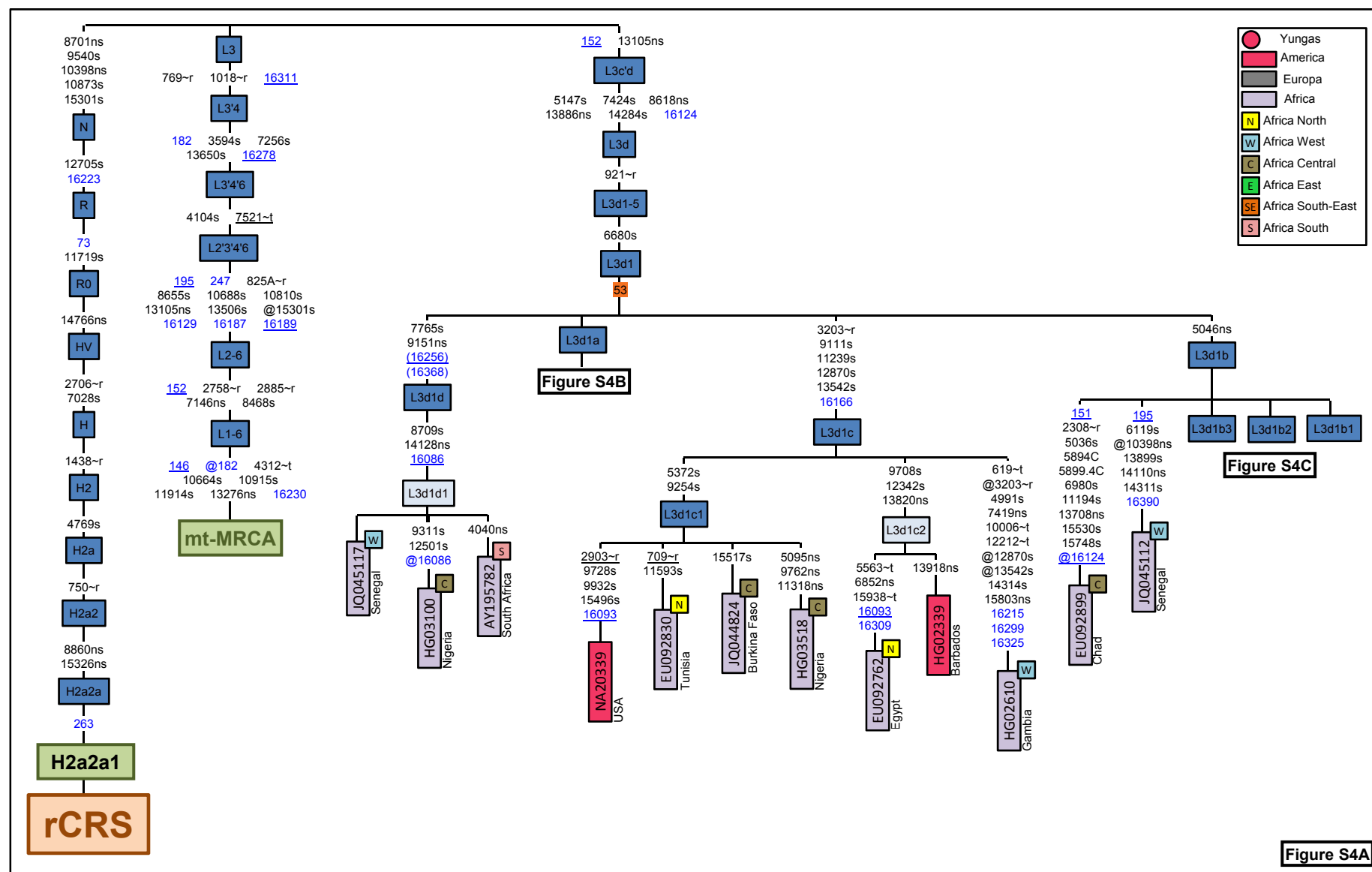

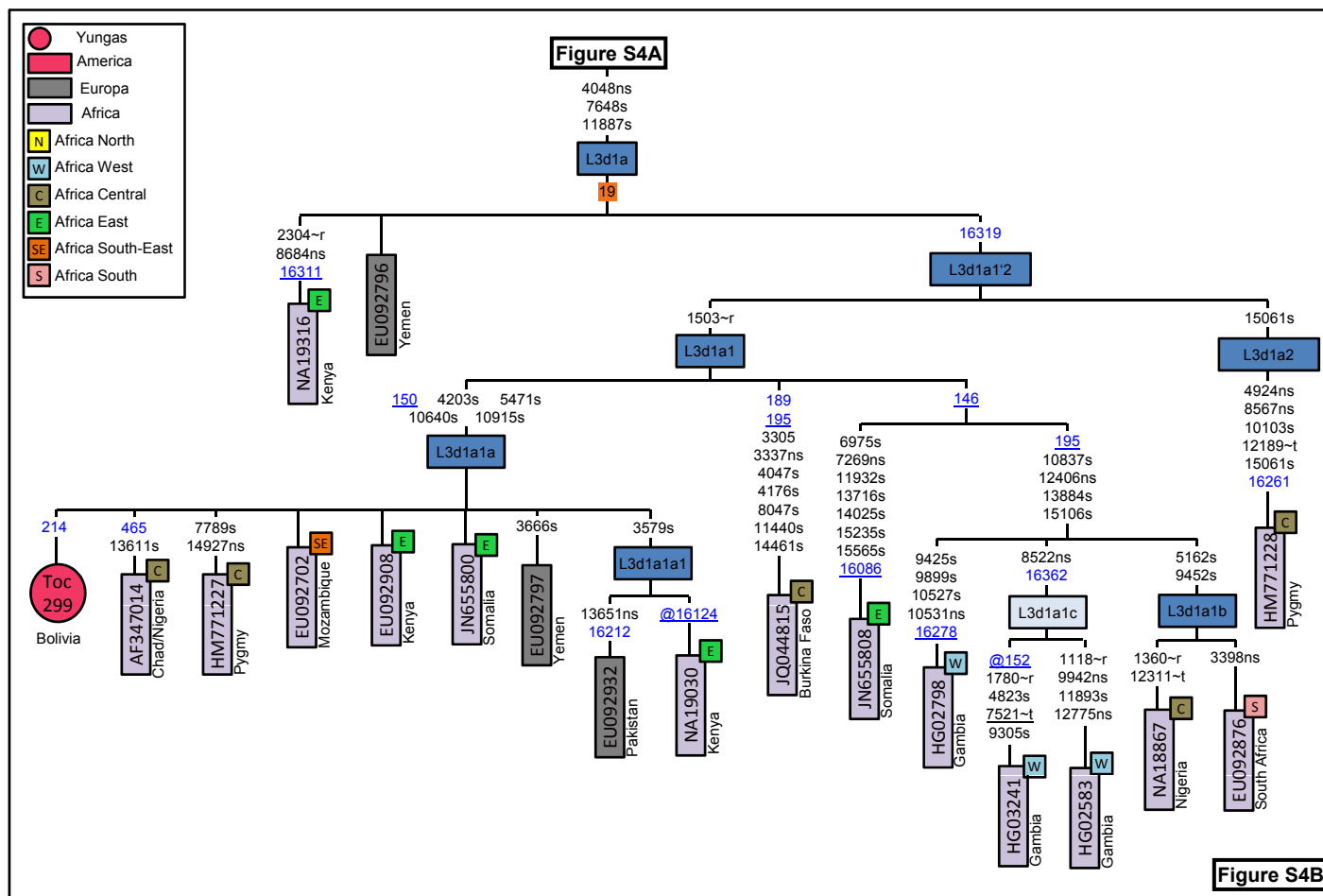

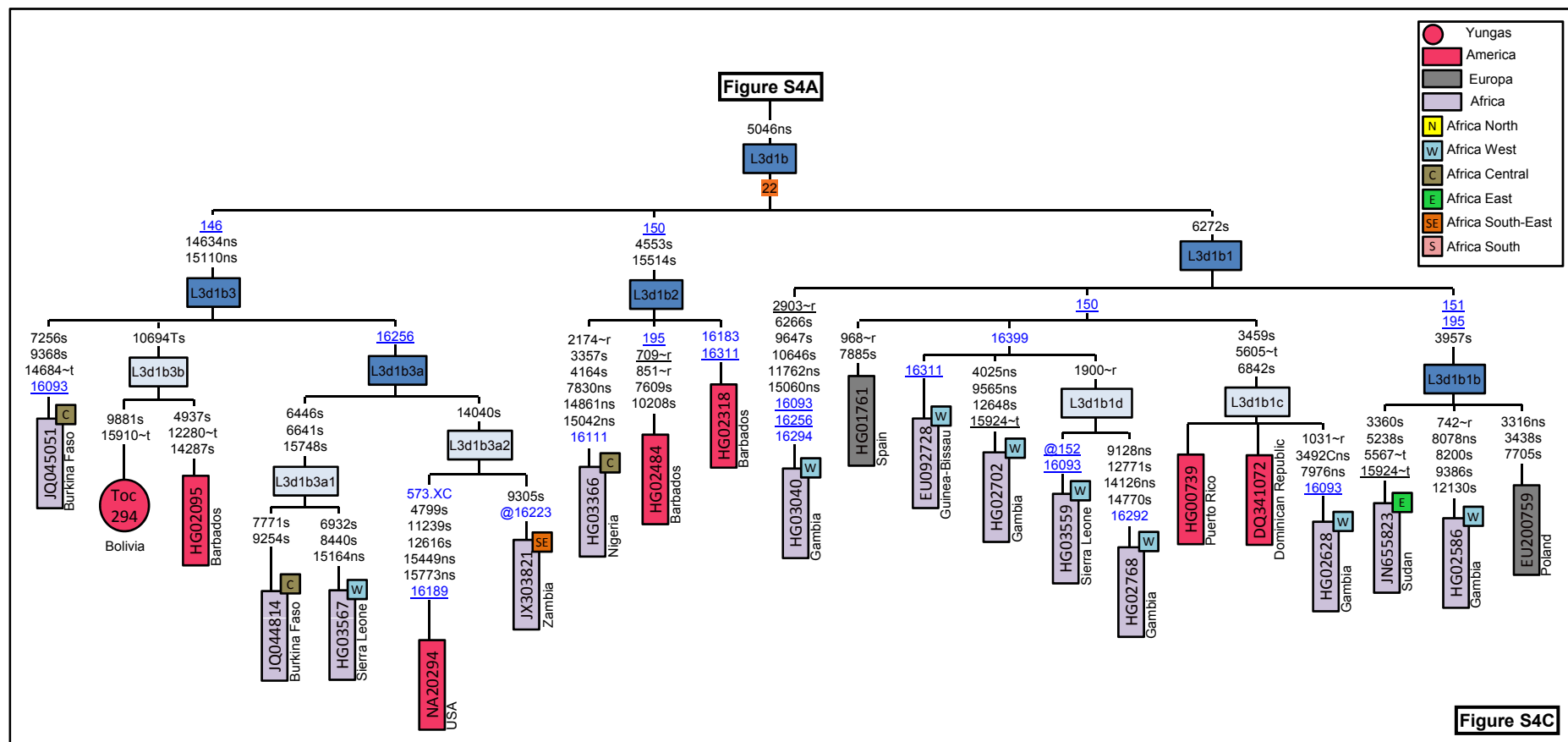

Supplement: S4 Fig — More details are given in legend of Fig 2. (PDF) [file pone.0134129.s004.pdf]
