# Supplementary material for: The Genomic Legacy of the Transatlantic Slave Trade in the Yungas Valley of Bolivia
Source: PLoS One. 2015 Aug 11;10(8):e0134129. doi: 10.1371/journal.pone.0134129 (PMC4532489; doi:10.1371/journal.pone.0134129)
Supplement: S1 Text — (DOC) [file pone.0134129.s012.doc]

**Text S1**

Notes on the Transatlantic Slave Trade

The beginning of the TAST can be traced back to the onset of the 15^th^ century when the first Portuguese landed on the sub-Saharan African coast . Although the main interest of the Portuguese at that time was gold, they also integrated themselves into the already existing African slave market within the continent. In the beginning, Portuguese traders sent enslaved people mainly as domestic servants to the European continent, but later also to the sugar plantations on the Atlantic Islands off the coast of West Africa when the Portuguese established intense trade relationships with the Kingdom of Congo. However, the simultaneous conquest of the Americas by the Portuguese and the Spaniards, and later by other Europeans, intensified the demand for free labor. Exploitation of the indigenous workforce in the New World was limited partly because the population of Native Americans suffered high mortality rates due to their lack of prior exposure to infectious disease (mainly smallpox, measles, and cholera). In addition to this, workforce demands within Spain and Portugal were very high resulting in high wages for Iberian workers, which in turn meant employment of Iberians in the New World was non-economical and not affordable. Taken together, the labor situation in the Americas and Europe, as well as the opening up of the TAST on the West African Coast by the Portuguese, jointly promoted an intense forced migration of enslaved Africans to the New World .

According to historical records, the Spaniards were the first to have the capital to transport enslaved people from Africa to their colonies in the New World, in the beginning mainly to the viceroyalty of Peru, but also to Mexico. The demand for African forced labor, however, increased in Spanish America in the second half of the 16^th^ century after the discovery of rich silver deposits in the mountainous Andes around Potosí in Upper Peru (present-day Bolivia) . Thus, the first African people in Potosí are documented from 1549 onwards . Most Africans were forced to work in the Royal Mint (“Casa de la Moneda”), which operated in Potosí until 1773, but some were also domestic servants . The Royal Mint contributed to a great extent to the economic boom of the Spanish colony, but enslaved Africans, who had to work and live under very harsh conditions, were excluded from the increasing economic advancement and suffered high mortality rates .

In the course of the 18^th^ and 19^th^ centuries, descendants of the first enslaved Africans in Potosí migrated northwards from the highland mining regions to the tropical Yungas valley. Here, the Spanish colonists established the so-called “hacienda system” — usually conceived as landed estates owned by colonists and developed as profit-making enterprises linked to national and international markets. Haciendas were maintained by forced slave labor and commonly owned by absentee estate holders. Enslaved people, ‘African descendants’ together with local Native Americans, were mainly working on coca, but also on coffee and sugar plantations . After the transition from colony to republic in 1825 and the first Bolivian Constitution, new slavery was abolished and enslaved people were allowed to purchase their freedom for a high price; however, protests by estate holders hindered the abolition of slavery .

Thus, the situation for ‘African-descendant’ people in the Yungas valley being forced to work did not change immediately after the colonial period. Only in 1952, after the agrarian reform in Bolivia, the “hacienda system” and thus slavery were abolished . Henceforth, ‘African-descendant’ people were free from hacienda labor but, nonetheless, the majority remained in the Yungas valley and occupied former estates .

**References**

1. Klein HS (2010) The Atlantic Slave Trade (New approaches to the Americas). Cambridge: Cambridge University Press.

2. Davies CEB (2008) Encyclopedia of the African diaspora: origins, experiences, and culture. Santa Barbara, California: ABC-CLIO. 1269 p.

3. Lipski JM (2006) Afro-Bolivian Spanish and Helvécia Portuguese: semi-creole parallels. Papia 16: 96-116.

4. Lipski JM (2006) Afro-Bolivian language today: the oldest surviving Afro-Hispanic speech community. Afro-Hispanic Review 25: 179.
