# Supplementary material for: The Genomic Legacy of the Transatlantic Slave Trade in the Yungas Valley of Bolivia
Source: PLoS One. 2015 Aug 11;10(8):e0134129. doi: 10.1371/journal.pone.0134129 (PMC4532489; doi:10.1371/journal.pone.0134129)
Supplement: S2 Text — (DOC) [file pone.0134129.s013.doc]

**Text S2.**

Phylogeographic connections between Afro-Bolivian mtDNAs and other American locations

The networks of ‘Afro-Bolivian’ mitogenomes reveal some interesting connections between ‘Afro-Bolivians’ and other American locations. Afro-Bolivian #Toc294 forms sub-branch L3d1b3b together with one sample from Barbados. #Toc293, #Toc295, and #Toc304 constitute sub-branch L1c3b1a with one individual from Puerto Rico. #Toc289 and #Toc290 form L0a1b2 including three individuals from the US, while #Toc305 within L0a2a2a again joins three samples from the U.S. but also one sample from the Dominican Republic.

The British colony Barbados became the final destination for many enslaved African people when the Dutch introduced sugar production on the island, leading to the transportation over 50,000 slaves by the end of the 17^th^ century. Barbados became the largest sugar producer for the British colonists before it was overtaken by Jamaica in the 1730s. A historical connection between the British and Spanish empires existed when Britain was allowed to trade slaves in Spanish America, thus introducing approximately 75,000 enslaved Africans to Buenos Aires, the Panamanian isthmus and Cartagena, but mostly to Upper Peru . According to the TAST database, most enslaved Africans who embarked on British ships came from the Bight of Biafra (31.2%) followed by the Gold Coast (19.3%), West-Central Africa (15.5%) and the Bight of Benin (9.3%), and these proportions are is reflected in the slaves who disembarked proportions on Barbados. Of these, most came from the Bight of Biafra (23.7%), followed by the Gold Coast (22.5%), the Bight of Benin (17.0%), and West-Central Africa (11.0%) . Isotopic analyses of skeletal remains in Barbados found African-born individuals that most probably originated from three different regions in Africa, including the Gold Coast and Senegambia . It is possible that enslaved people who arrived in Spanish America or Barbados, respectively, might have originated from the same geographical region in Africa where the British concentrated their trade.

The United States was also a British colony with slavery centered especially in South Carolina, Georgia and in the Chesapeake Bay, including Virginia, Maryland and part of North Carolina. The Chesapeake Bay was a major tobacco production area while the Georgia-South Carolina region developed to be a major rice producer . In Mainland North America, most enslaved Africans came first and foremost from West-Central Africa (26%), followed by Senegambia (20.3%), the Bight of Biafra (16.2%), the Gold Coast (11%), and Sierra Leone (11.1%) . The increased number of slaves from Senegambia, and Sierra Leone, may be explained by the merchants’ preferences for slaves from the Grain Coast (modern Senegal, Gambia, Sierra Leone, west Liberia) due to their rice cultivating skills . Nevertheless, since Great Britain was allowed to trade slaves in Spanish America , enslaved Africans in both the Spanish and British colonies that travelled on British ships to the United States and to Spanish America might have had similar geographical roots in Africa.

Puerto Rico, in contrast, was a Spanish colony and reached a peak of African enslavement in the 1830s with a slave population of approximately 42,000 Africans. Even after the British and the United States had abandoned the slave trade, shipments of enslaved Africans continued to arrive to Cuba and Brazil, and also to Puerto Rico . Most slaves who disembarked in Puerto Rico came from the Bight of Biafra (40%) followed by West-Central Africa (32.1%), Sierra Leone (11%) and Southeast Africa (4.3%). Southeast Africa became increasingly important towards the end of the TAST. The largest number of enslaved Africans arrived in Puerto Rico during the peak of the TAST from 1766 to 1770 (57.8%); however, the second largest number of slaves arrived between 1836 and 1840 (10.9%) .

The importation of enslaved Africans to the Spanish colony of Santo Domingo (nowadays Dominican Republic) began as early as 1502 . On the other side of Hispaniola, lies Saint-Domingue (nowadays Haiti), which developed to become a giant sugar plantation within the French colony during the TAST . Of those Africans who ended up in Santo Domingo most came from West-Central Africa (47.0%), followed by the Bight of Biafra (16.1%) and the Bight of Benin (12.4%) . Similarly, in Saint-Domingue, most African slaves came from West-Central Africa (46.1%) and the Bight of Benin (24.7%). However, in both parts of Hispaniola slave arrivals from Southeast Africa were minimal according to the Transatlantic Slave Voyages database (Santo Domingo: unknown, Saint-Domingue: 3.3%).

Based on genetic and historical research no clear and definite conclusion can be reached about how ‘Afro-Bolivian’ populations might be linked to other American populations. Further research is necessary to disentangle the routes followed by the ancestors of ‘Afro-Bolivans’, as well as of other African-descendant American populations.

References

1. Klein HS (2010) The Atlantic Slave Trade (New approaches to the Americas). Cambridge: Cambridge University Press.

2. Eltis D (2008) A brief overview of the Trans-Atlantic Slave Trade.” Voyages: The Trans-Atlantic Slave Trade Database.

3. Schroeder H, O'Connell TC, Evans JA, Shuler KA, Hedges RE (2009) Trans-Atlantic slavery: isotopic evidence for forced migration to Barbados. Am J Phys Anthropol 139: 547-557.

4. Littlefield DC (1991) Rice and Slaves: Ethnicity and the Slave Trade in Colonial South Carolina; Illinois Uo, editor: Blacks in the New World.

5. Hall EJ (2005) Slavery and African Ethnicities in the Americas: Restoring the links. USA: The University of North Carolina Press.
