# Supplementary material for: The Genomic Legacy of the Transatlantic Slave Trade in the Yungas Valley of Bolivia
Source: PLoS One. 2015 Aug 11;10(8):e0134129. doi: 10.1371/journal.pone.0134129 (PMC4532489; doi:10.1371/journal.pone.0134129)
Supplement: S3 Text — (DOCX) [file pone.0134129.s014.docx]

**Text S2**

Minimal haplotype searches in the Y-haplotype reference database (YHRD; <http://yhrd.org>) of those Y-STR haplotypes that have been classified as belonging to an African haplogroup according to Y-SNP information (**Table S1**). The last two maps correspond to two African Y-STR profiles that are from non-Yungas Bolivia and were analyzed also in Cárdenas et al. [[1](#_ENREF_1)].

**Sample 295Tocaña (Tocaña; Andean): haplogroup BR**


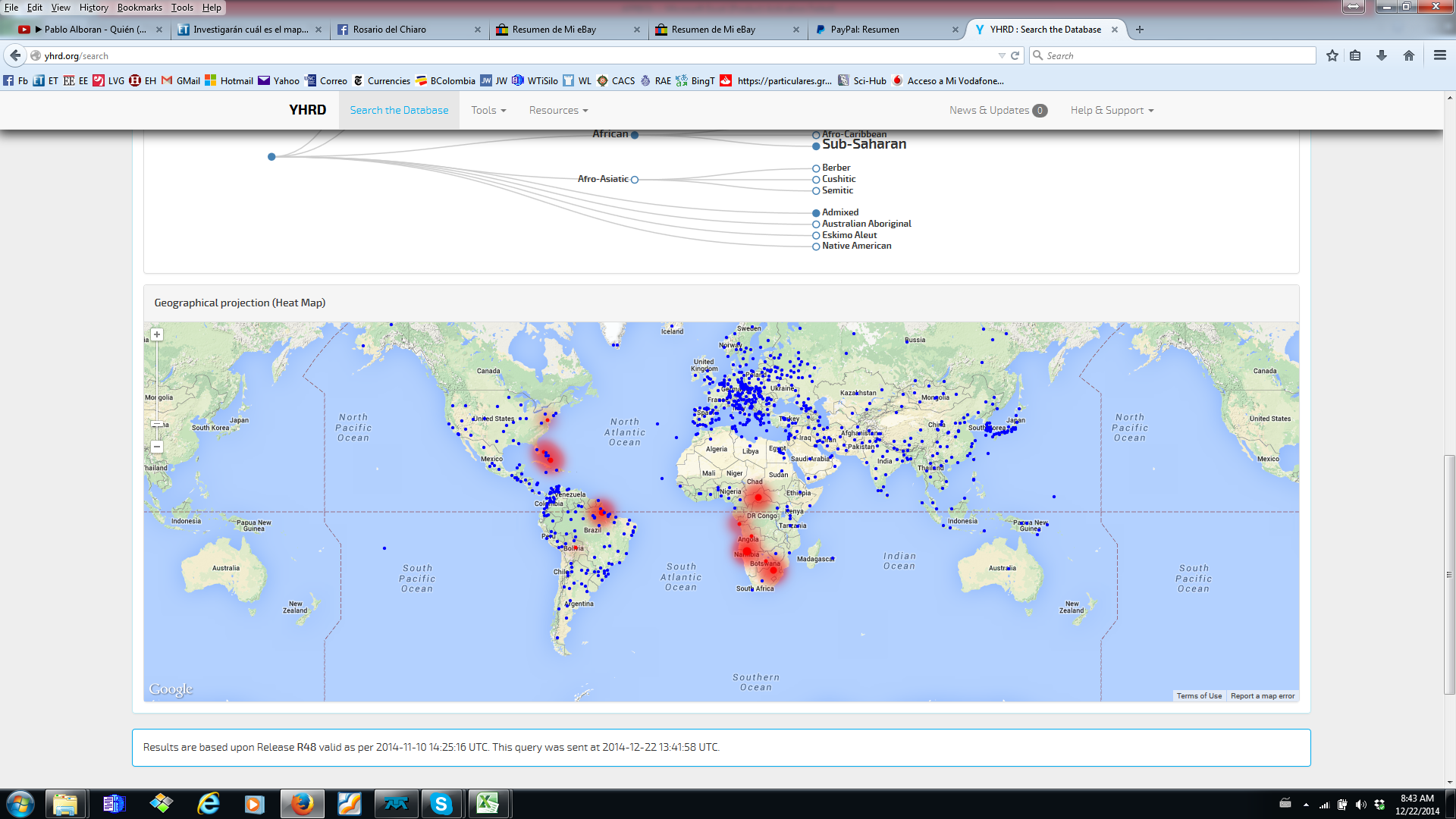


**Sample Tocaña291 (Tocaña; Andean): haplogroup E**


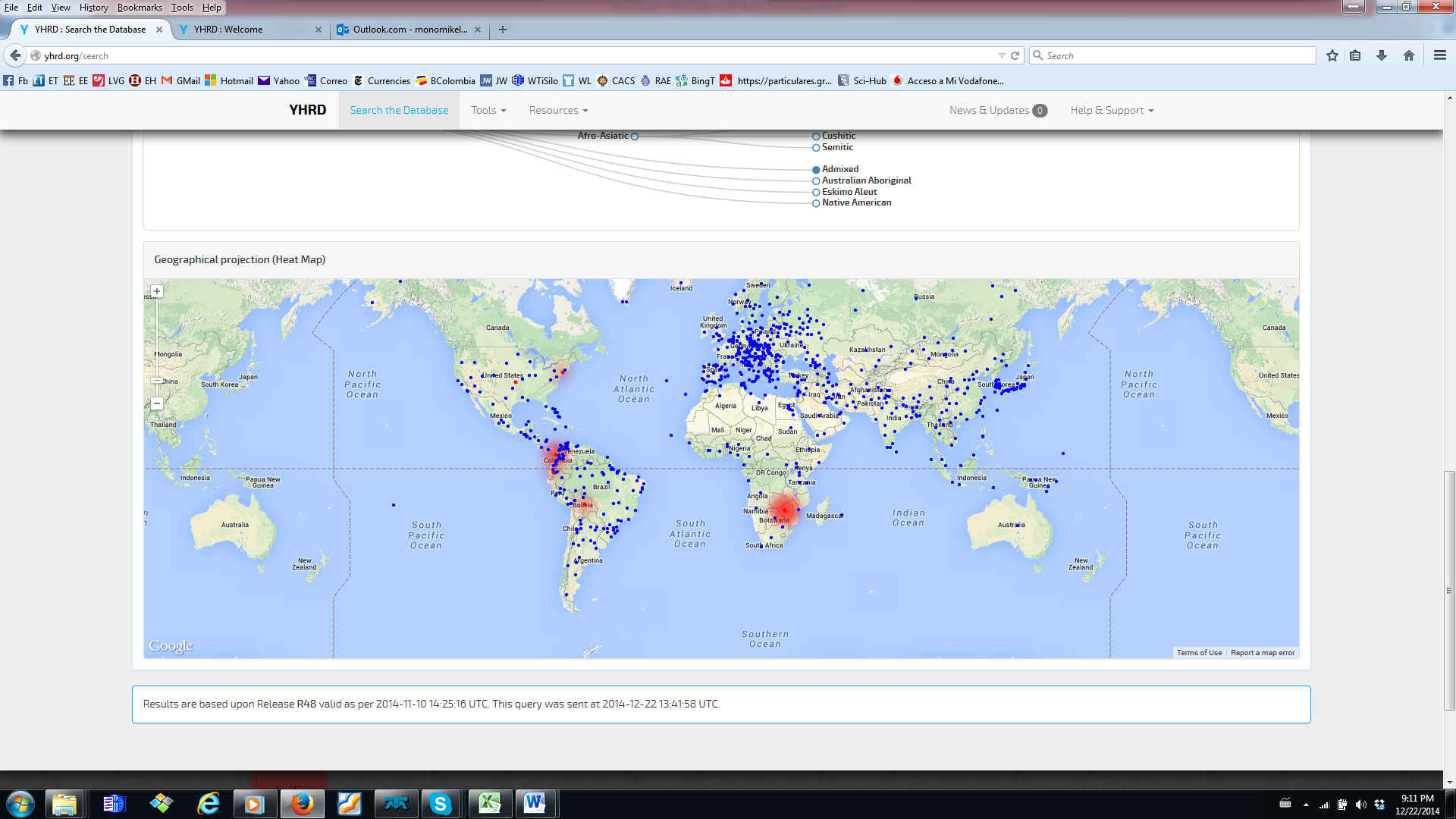


**Tocaña292 (Tocaña; Andean): haplogroup E**


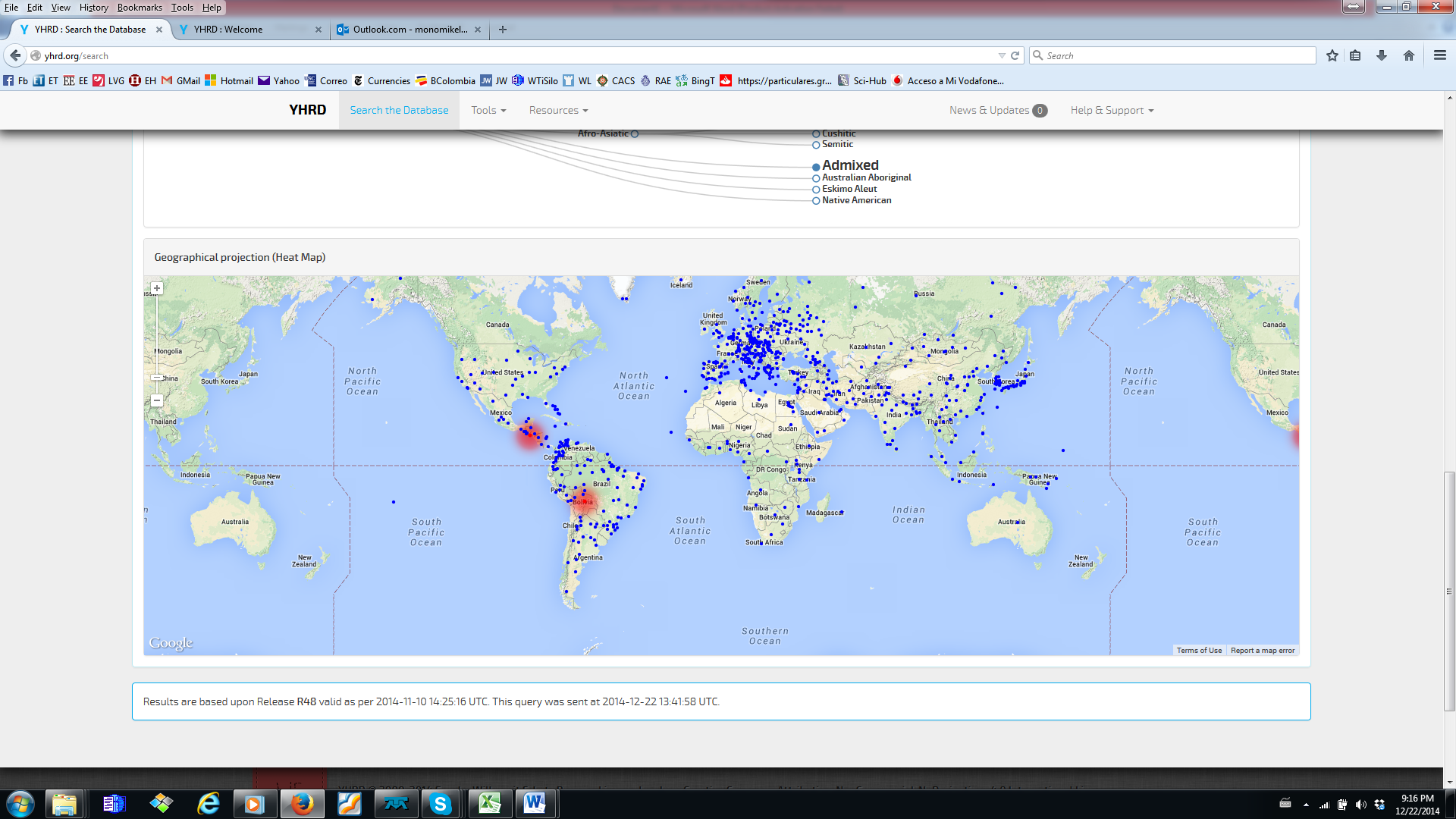


**Sample Tocaña294 (Tocaña; Andean): haplogroup E1b1b1***


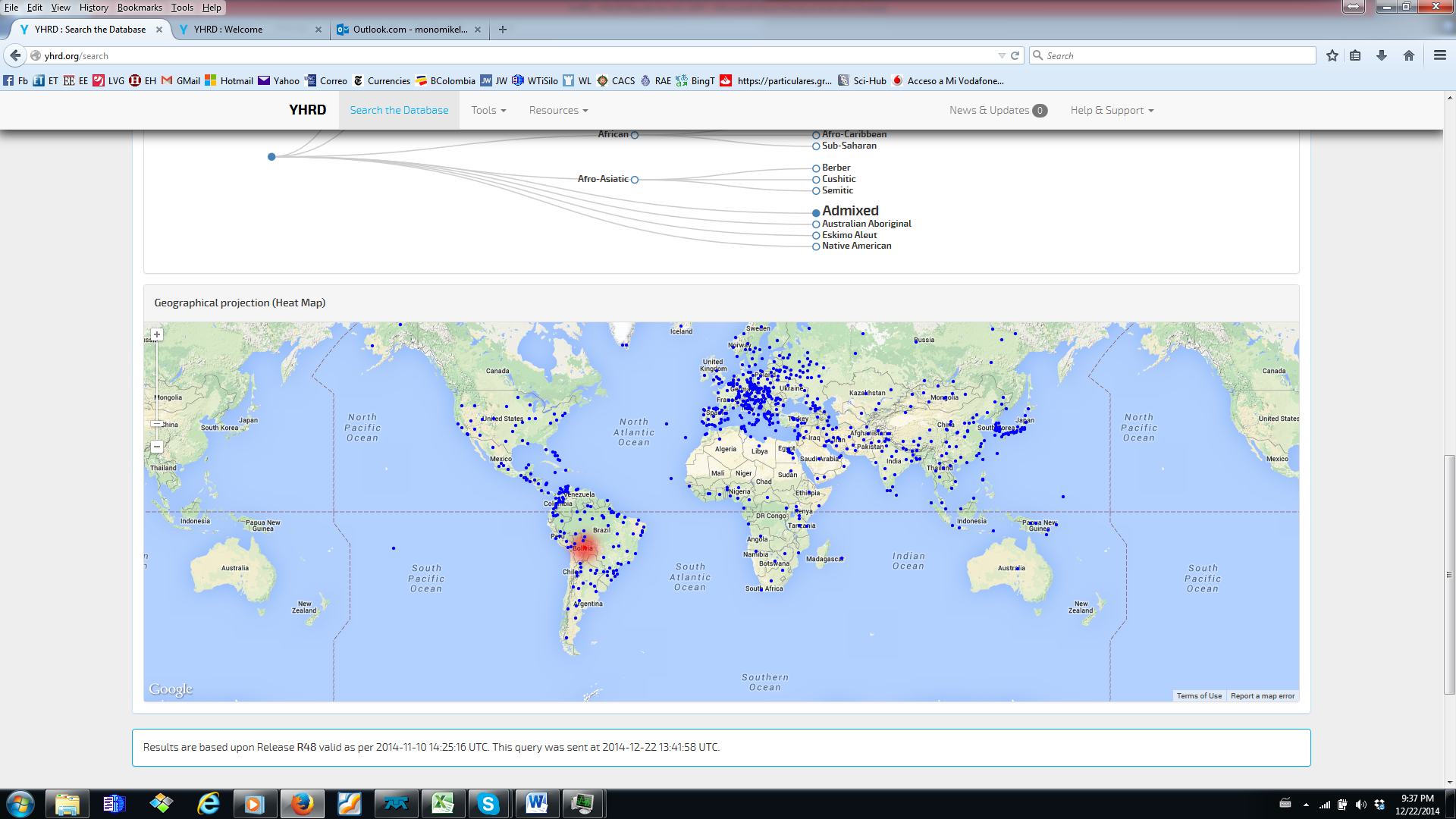


**Sample SC257 (Santa Cruz in Llanos): haplogroup E***


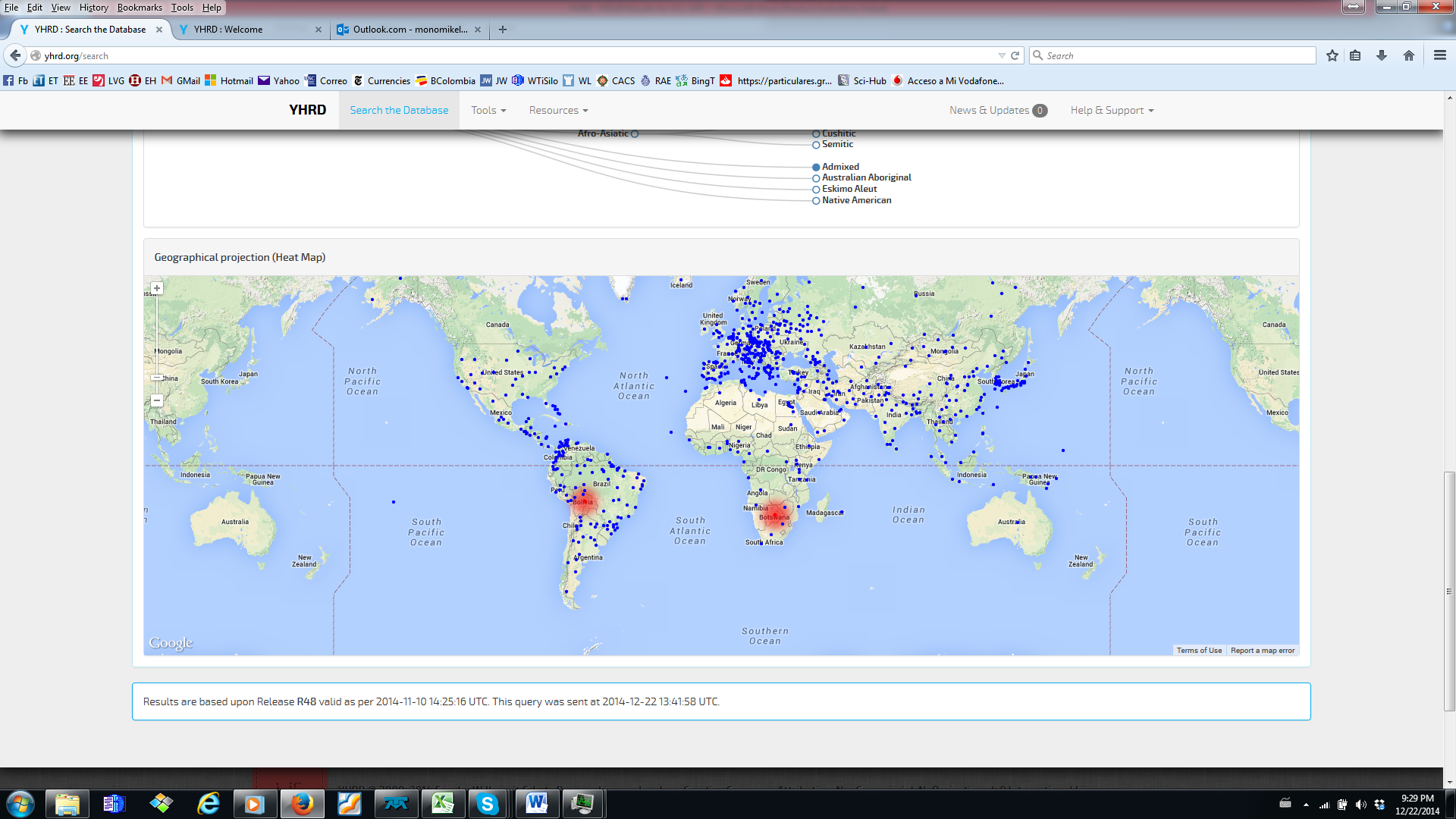


**Sample SC219 (Santa Cruz in Llanos): haplogroup E***


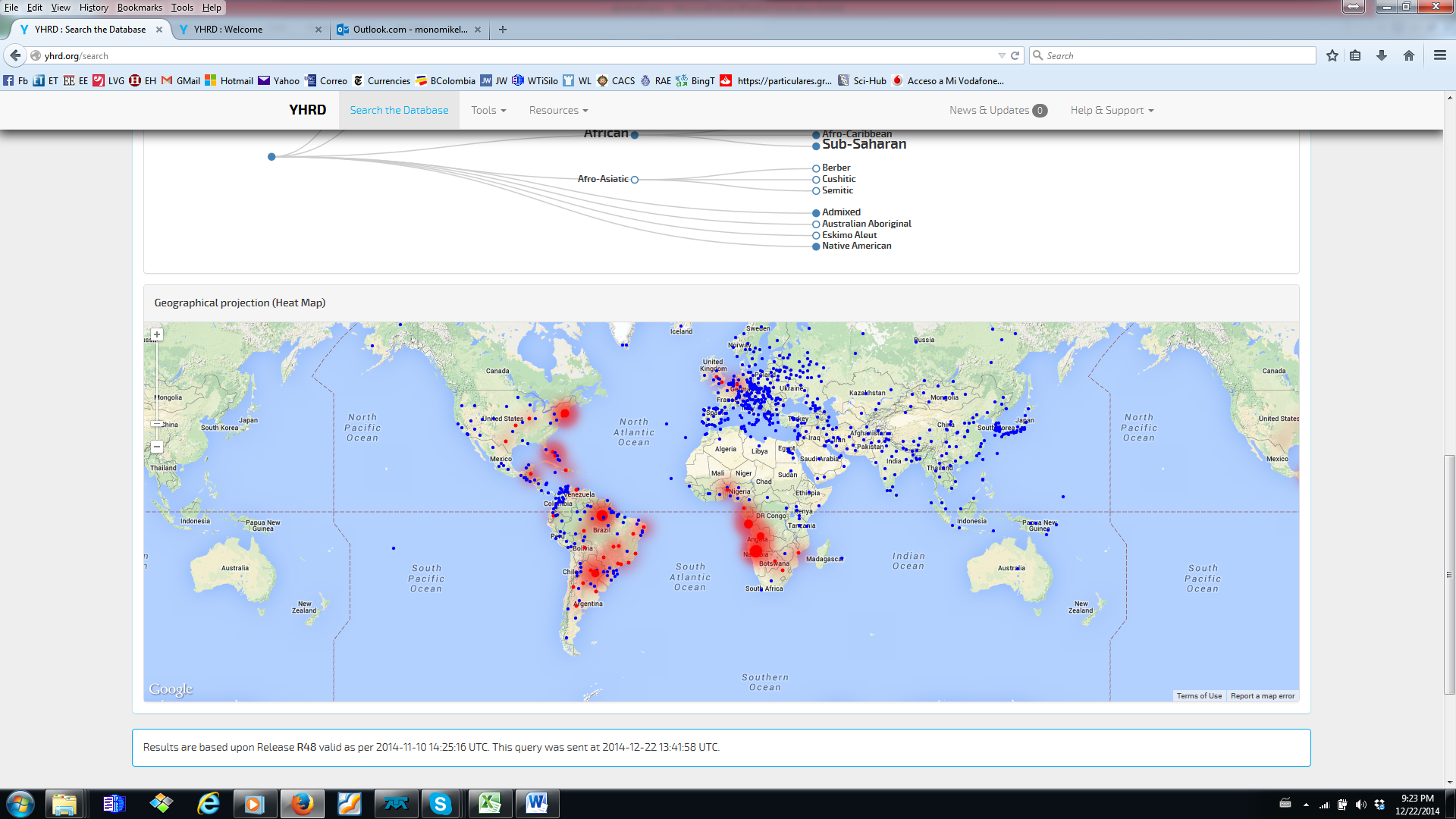


**References**

1. Cárdenas JM, Heinz T, Pardo-Seco J, Álvarez-Iglesias V, Taboada-Echalar P, et al. (2014) The multiethnic ancestry of Bolivians as revealed by the analysis of Y-chromosome markers. Forensic Sci Int Genet 14: 210-218.
