# Supplementary material for: The Genomic Legacy of the Transatlantic Slave Trade in the Yungas Valley of Bolivia
Source: PLoS One. 2015 Aug 11;10(8):e0134129. doi: 10.1371/journal.pone.0134129 (PMC4532489; doi:10.1371/journal.pone.0134129)
Supplement: S4 Text — (DOCX) [file pone.0134129.s015.docx]

**Text S4.**

Arab slave trade and its connections with the TAST

Even though many Muslims objected to the enslavement of free Muslims to the New World and across the Sahara, it has been reported that up to 40% of all enslaved people who were forced to leave Africa between 1500 and 1800 can be attributed to the Arab slave trade [[1](#_ENREF_1)]. The phylogenetic proximity of ‘Afro-Bolivian’ mtDNAs from Tocaña to Southeast and East Africa and also to the Middle East, might reflect a connection between the Arab slave trade and the TAST.

From 1400 to 1600 slavery within Africa was most prominent along the southern borders of the Sahara Desert, the shores of the Red Sea, and the East African coast and was predominantly controlled by Muslims [[1](#_ENREF_1)]. The major external slave markets were concentrated in North Africa and in the Middle East. However, after the arrival of Europeans, slavery expanded within Africa by distributing further enslaved people within Africa but also to the Americas. There were six major routes, which were also interconnected, that linked sub-Saharan Africa to North and East Africa. For example, Muslim traders connected ancient Ghana with Morocco [[1](#_ENREF_1)] (note that one Tocaña [#Toc294] forms one sub-branch, L3d1b3, with two individuals from Burkina Faso). Estimates about numbers of slaves might be less accurate for the Arab slave trade than for the TAST, but it has been suggested that approximately 400,000 enslaved people departed from the Red Sea region, 1,950,000 from the Sahara, and 600,000 from East Africa between the years 1500 and 1800. Muslim traders were an important agent during the TAST. For example, on the Bight of Benin or ‘Slave Coast’, where the states were more centralized, Europeans were confined to the coast for trade and did not raid for slaves, as was the case in West-Central Africa where the states were more fragmented. Nonetheless, Muslim traders played a pivotal role in establishing a link between the far interior and the states that exported slaves [[1](#_ENREF_1)].

The slave trade in East Africa can be divided into three sectors: (i) trade that led to northern states such as Arabia, Persia, and India; (ii) trade to the Americas and the Mascarenes, and (iii) coastal trade. In the 18^th^ century, an estimated 400,000 individuals were displaced in the East African slave trade, with approximately two thirds who were destined for traditional markets in the Muslim world, and the rest sent to the Mascarene Islands or to the New World. Approximately 1,650,000 enslaved people were involved in this trade in the 19^th^ century. Half of them remained in East Africa and the other half were forced to migrate to the Americas or to other places in the Indian Ocean [[1](#_ENREF_1)]. East Africa became increasingly important for slave traders in the 19^th^ century, as pro-abolition efforts by the British were at first chiefly concentrated in West and West-Central Africa. After the British abolished the slave trade in 1807 and in its colonies in 1808, Great Britain forced the other European countries to stop slaving activities by stationing its navy along the African coast. However, the British effort to abolish slave trade *via* repeated attempts to negotiate with other European nations only became increasingly noticeable after 1840 on the Atlantic coast and even later in East Africa [[1](#_ENREF_1),[2](#_ENREF_2)].

Most enslaved Africans who left East/Southeastern Africa for the Americas embarked on ships in Mozambique. Here, the number of slaves destined for the Americas peaked at around 35,000 slaves between 1836 and 1840 [[3](#_ENREF_3)]. Mozambique became an especially important source of African slaves for Brazilians and the Portuguese [[4](#_ENREF_4)]. According to the TAST databasethe first enslaved Africans left Southeastern Africa in 1661, starting a process that culminated in the first half of the 19^th^ century [[3](#_ENREF_3)]. However, at least 1,536 people from the area around Congo and Angola, but also from Mozambique, are documented in Bolivia as early as 1535 [[5](#_ENREF_5)]. Southeastern Africa became the second most important slave supplier between 1811 and 1840 (after West-Central Africa) when the British expanded efforts to abolish slavery [[2](#_ENREF_2),[3](#_ENREF_3)]. From 1841 onwards, the number of slaves gradually decreased and, thus, Southeastern Africa was positioned in third place after West-Central Africa and the Bight of Benin with respect to slave numbers. About 112,800 slaves embarked on ships during Southeastern Africa’s peak from 1821 to 1830. Most enslaved Africans (70.6%) were forced to embark on ships headed to Brazil, but some also went to the Caribbean (19.9%) [[3](#_ENREF_3)].

The East African slave trade was more complex than its counterparts in Western and West-Central Africa. Slaves in East Africa were not only destined fortheTAST, but also for the Trans-Saharan trade and for the trade across the Red Sea and Indian Ocean [[1](#_ENREF_1)].

In Bolivia slavery was officially abolished only after the agrarian reform in 1952. After the emancipation of Bolivia from the Spanish colonists in 1825, attempts to realize the abolition of slavery failed several times [[6-8](#_ENREF_6)]. Given the prevailing importance of Southeastern Africa with respect to slave numberstowards the end of the TAST, and the late abolition of slavery in Bolivia, an East/Southeastern origin for slaves in Bolivia might be reasonable. Furthermore, the coastal trade along the East African coast might also explain the Middle Eastern origin of ‘Afro-Bolivians’. Most enslaved people at the end of the TAST were destined for Brazil, Cuba, and Puerto Rico [[2](#_ENREF_2)]. Major ports of arrival for enslaved Africans who reached Bolivia included ports in Brazil such as Recife, Salvador-Bahía, and Río de Janeiro. Therefore, enslaved Africans from East/Southeastern Africa might have arrived in present-day Bolivia *via* Brazil during this period.

In any case, these questions have to remain open given the shortage of historical and genetic information hitherto available. In spite of our efforts, we have not been able to find documentation pertaining to when the last enslaved Africans arrived in the area of present day Bolivia.

References

1. Lovejoy P (2000) Transformations in Slavery: A History of Slavery in Africa. Cambridge: Cambridge University Press.

2. Klein HS (2010) The Atlantic Slave Trade (New approaches to the Americas). Cambridge: Cambridge University Press.

3. Eltis D (2008) A brief overview of the Trans-Atlantic Slave Trade.” Voyages: The Trans-Atlantic Slave Trade Database.

4. Hall EJ (2005) Slavery and African Ethnicities in the Americas: Restoring the links. USA: The University of North Carolina Press.

5. Rodríguez R, J. (2001) The Afro populations of America's southern cone: organization, development, and culture in Argentina, Bolivia, Paraguay, and Uruguay; S.S. W, editor. Oxford, England: Towman & Littlefield Publishers, Inc.

6. Davies CEB (2008) Encyclopedia of the African diaspora: origins, experiences, and culture. Santa Barbara, California: ABC-CLIO. 1269 p.

7. Lipski JM (2006) Afro-Bolivian spanish and helvécia portuguese: semi-creole parallels. Papia 16: 96-116.

8. Lipski JM (2006) Afro-Bolivian language today: the oldest surviving Afro-Hispanic speech community. Afro-Hispanic Review 25: 179.
